# Dynamic prostate cancer transcriptome analysis delineates the trajectory to disease progression

**DOI:** 10.1038/s41467-021-26840-5

**Published:** 2021-12-02

**Authors:** Marco Bolis, Daniela Bossi, Arianna Vallerga, Valentina Ceserani, Manuela Cavalli, Daniela Impellizzieri, Laura Di Rito, Eugenio Zoni, Simone Mosole, Angela Rita Elia, Andrea Rinaldi, Ricardo Pereira Mestre, Eugenia D’Antonio, Matteo Ferrari, Flavio Stoffel, Fernando Jermini, Silke Gillessen, Lukas Bubendorf, Peter Schraml, Arianna Calcinotto, Eva Corey, Holger Moch, Martin Spahn, George Thalmann, Marianna Kruithof-de Julio, Mark A. Rubin, Jean-Philippe P. Theurillat

**Affiliations:** 1 Faculty of Biomedical Sciences, Institute of Oncology Research, USI, Bellinzona, TI 6500 Switzerland; 2 Computational Oncology Unit, Department of Oncology, Istituto di Richerche Farmacologiche ‘Mario Negri’ IRCCS, 20156 Milano, Italy; 3 Bioinformatics Core Unit, Swiss Institute of Bioinformatics, TI 6500 Bellinzona, Switzerland; 4 Department of Biomedical Research, University of Bern, 3008 Bern, Switzerland; 5 Oncology Institute of Southern Switzerland, Bellinzona, TI 6500 Switzerland; 6 Urology Department, Ente Ospedaliero Cantonale, Bellinzona, TI Switzerland; 7 Faculty of Biomedical Sciences, University of Southern Switzerland (USI), TI 6900 Lugano, Switzerland; 8 Institute of Surgical Pathology, University Hospital Basel, 4031 Basel, Switzerland; 9 Department of Pathology, University Hospital Zurich, 8091 Zurich, Switzerland; 10 Department of Urology, University of Washington, Seattle, WA 98195 USA; 11 Lindenhofspital Bern, Prostate Center Bern, 3012 Bern, Switzerland; 12 Department of Urology, Inselspital, Bern University Hospital, 3010 Bern, Switzerland; 13 Bern Center for Precision Medicine, University of Bern and Inselspital, 3012 Bern, Switzerland

**Keywords:** Tumour heterogeneity, Prostate cancer

## Abstract

Comprehensive genomic studies have delineated key driver mutations linked to disease progression for most cancers. However, corresponding transcriptional changes remain largely elusive because of the bias associated with cross-study analysis. Here, we overcome these hurdles and generate a comprehensive prostate cancer transcriptome atlas that describes the roadmap to tumor progression in a qualitative and quantitative manner. Most cancers follow a uniform trajectory characterized by upregulation of polycomb-repressive-complex-2, G2-M checkpoints, and M2 macrophage polarization. Using patient-derived xenograft models, we functionally validate our observations and add single-cell resolution. Thereby, we show that tumor progression occurs through transcriptional adaption rather than a selection of pre-existing cancer cell clusters. Moreover, we determine at the single-cell level how inhibition of EZH2 - the top upregulated gene along the trajectory – reverts tumor progression and macrophage polarization. Finally, a user-friendly web-resource is provided enabling the investigation of dynamic transcriptional perturbations linked to disease progression.

## Abstract (web summary)

Transcriptional changes during prostate cancer progression are not yet fully understood. Here, the authors integrate a transcriptomics atlas of prostate cancer and validate it with preclinical models and single-cell RNA-seq, revealing the role of EZH2 and macrophage polarisation in tumour progression.

## Introduction

Many decades of research have established the fundamental understanding of cancer as an anarchistic proliferation and dissemination of cells caused by acquired mutations in key driver genes^1^. During the past decade, the most common cancer types have been extensively characterized for alterations in the tumor DNA sequence^2^. While these studies have been initially conducted on primary cancer tissues, more recent clinical studies have also included biopsies from metastatic disease^3–9^. Because of the binary nature of DNA sequence alterations (mutated versus non-mutated), mutation frequencies can be readily compared across studies and enable the nomination of drivers intimately linked to disease progression and outcome^10,11^. That said, the plethora of complex genetic alterations largely complicates a quantitative assessment of the transformed phenotype.

The assessment of gene expression may provide a more complete and quantitative measure of the biological processes related to disease progression. Most transcriptomic studies have been thus far conducted on primary tumors^12,13^. However, multiple efforts have been dedicated in recent years to the characterization of metastatic disease for a few tumor types, including prostate cancer, opening the possibility to assess transcriptional changes along with disease progression in a systematic manner^11,14–17^.

Nevertheless, this approach requires the accurate integration of multiple datasets across studies to overcome the issue of introducing dataset-specific features, often referred to as batch effects. The substantial amount of nonbiological artifacts introduced both by RNA-sequencing (RNA-seq) library generation techniques and by the exploitation of different quantification algorithms are among the difficulties that can emerge in the attempt of nominating a trajectory of prostate cancer disease progression by inferring dynamic transcriptional changes from a large integrated cohort.

Here, we provide a framework to overcome these issues and enable the accurate quantitative integration of RNA-seq data from over 1000 clinical tissues ranging from normal prostate tissue to primary prostate cancer (PNPCa) and metastatic castration-resistant (CR) prostate cancer (CRPC). The harmonized Prostate Cancer Transcriptome Atlas provides a unique resource to mine transcriptional changes related to different disease stages. Using this resource, we characterize the trajectory to disease progression and functionally validate our findings in patient-derived xenograft (PDX) models at the single-cell level. Finally, we show how our Prostate Cancer Transcriptome Atlas can infer or validate new therapeutic avenues for cancer patients.

## Results

### Generation of the Prostate Cancer Transcriptome Atlas

**Fig. 1:**
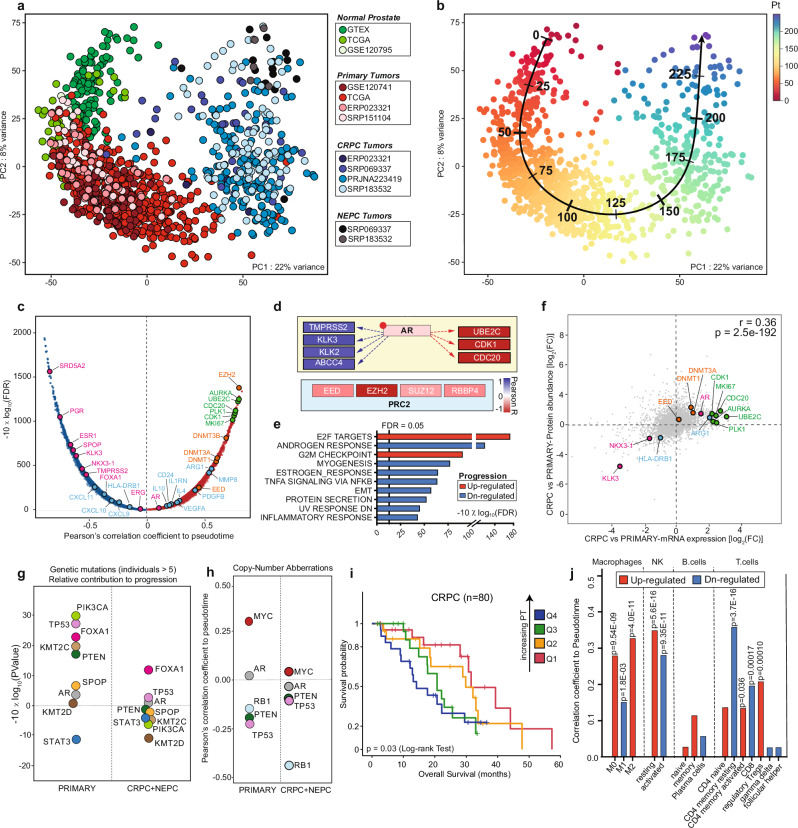
Trajectory to prostate cancer progression. **a** Principal component analysis (PCA) of pan-prostate cancer transcriptomes obtained from the indicated studies of normal (shades of green), primary (shades of red), castration-resistant (CRPC, shades of blue), and neuroendocrine prostate cancer (NEPC, shades of gray). See Source data file. **b** Unbiased trajectory analysis identifies the path to disease progression. Quantification of the path is indicated by inferred pseudotime. See Source data file. **c** Plot representing the correlation between mRNAs and pseudotime inferred along the trajectory. Positively correlated genes are depicted in red while negatively correlated genes are depicted in blue. Polycomb-repressive complex-related genes highlighted in orange, cell cycle-related genes in green, immune response in light blue, and AR signaling in magenta. *X*-axis: Pearson’s correlation coefficient between mRNAs and pseudotime; *Y*-axis: the associated significance adjusted for false discovery rate (FDR) and expressed in the form of −10 × log 10(FDR). See Source data file. **d** Schematic representation of gene-expression changes in *AR*-regulated target genes related to cell differentiation and proliferation and PRC2 components along the trajectory. Genes are enclosed in boxes, whose color is associated to the correlation coefficients between mRNA expression and pseudotime, and are depicted in the indicated color scale. **e** Gene-set enrichment analysis performed on genes ranked for their Pearson’s coefficient as determined by the correlation between mRNA expression and pseudotime inferred from the trajectory. Increasing pseudotime results in an increase of cell cycle-related genes and concomitant downregulation of androgen-responsive genes. Upregulated: red; downregulated: blue. See Source data file. **f** Scatterplot revealing Pearson’s correlation coefficient and associated *P* value between mRNAs and protein abundances, expressed in the form of fold change (log-scale) between CRPCs and primary tumors. Polycomb-repressive complex-related genes highlighted in orange, cell cycle-related genes in green, immune response in light blue, and AR signaling in magenta. See Source data file. **g** *P* values associated to Pearson’s correlation coefficients expressed in form of −10 × log 10(*P* value) (FDR-adjusted). Coefficients were determined for the correlation between somatic mutations (0: wild type; 1: non-synonymous mutation) and inferred pseudotime along the trajectory. To dissect the relative impact on disease progression at different stages, coefficients were computed separately in primary and CRPC/NEPC samples. Only recurrently mutated genes (at least in six individuals) were taken into account. PIK3CA (green); TP53 (pink); PTEN (dark green); SPOP (orange); AR (gray); KMT2D (brown); KMT2C (light brown); STAT3 (blue). See Source data file. **h** Computed Pearson’s correlation coefficients between samples’ numeric copy-number status (−2: homozygous deletion; −1: heterozygous deletion; 0: wild type; 1: gain; 2: amplification) and inferred pseudotime, stratified for primary and metastatic tumors (CRPC, NEPC). MYC (red); AR (gray); RB1 (light blue); PTEN (dark green); TP53 (pink). **i** Kaplan–Meier curve for disease-free survival in CRPC patients stratified according to pseudotime using a four-tiered scoring system (quartiles: Q1, Q2, Q3, and Q4) reveals a significant association of higher pseudotime with impaired survival. Source: cBioportal (SU2C/PCF Dream Team, *PNAS* 2019). *P* value was determined by using log-rank test. Q1 (red, *n* = 20); Q2 (orange, *n* = 20); Q3 (green, *n* = 20); Q4 (blue, *n* = 19). For one patient, two metastatic samples were available. We discarded the sample with the lower pseudotime. See Source data file. **j** Histograms depicting the correlation between the inferred abundance of the indicated immune cell populations (as determined by Cibersortx) and pseudotime. *P* values associated with Pearson’s correlation coefficients were adjusted for multiple testing using false discovery rate (FDR) and reported on top of the bars. Red: positive correlation; blue: negative correlation. See Source data file.

To nominate gene-expression changes related to disease progression, we re-processed and integrated high-throughput transcriptional datasets from 13 different studies, constituting thus far the most comprehensive compendium of the disease (Supplementary Fig. 1A and Supplementary Data 1)^11,16–24^. The resulting principal component analysis (PCA) showed that samples’ position at a given disease stage largely overlapped with another regardless of their origin. In contrast, samples from distinct disease stages differed in localization (Fig. 1a). An appreciable “batch effect” related to the hybrid capture sequencing (HCS) technique was detected and subsequently corrected (Supplementary Fig. 1B).

Gene-set enrichment analysis (GSEA) of the first two principal components (PCs) revealed that PC1 correlated with enhanced proliferation, while PC2 anticorrelated with canonical AR signaling (Supplementary Fig. 1C, D). Moreover, PC3 separated cancers harboring truncal mutations in *SPOP* and *FOXA1* from the ones harboring gene fusions involving ETS family transcription factors (Supplementary Fig. 1E)^25–28^. Additional PCs accounting individually for <4% of the total variance did not reveal any association with tumor cell-specific features. Importantly, the stromal contribution was well represented by PC5 and to a much lesser extent associated with PC1–4 (Supplementary Fig. 1F–H and Supplementary Data 2). The latter indicates that the positioning of tissue samples in PC1–4 is only slightly influenced by the tumor purity.

### Trajectory analysis quantifies the path to disease progression

We applied trajectory inference analysis to characterize disease progression. The approach identified the path to disease progression and assigned a pseudotime to each sample that describes the advancements along this specific path (Fig. 1b). Because PC3 was mainly influenced by truncal prostate cancer driver mutations, its addition to the trajectory inference analysis did not affect the assigned pseudotime (Supplementary Fig. 1I, J). Subsequently, we assessed corresponding gene-expression changes to the initial two-dimensional trajectory (Fig. 1c). Among the most upregulated genes, we noticed key genes encoding for chromatin remodelers, which mediate gene silencing during development, such as DNA methyltransferases (DNMTs) and members of the polycomb-repressive complex-2 (PRC2)^29^. Most importantly, the PRC2 member *EZH2* emerged as the top upregulated gene, corroborating its previously suggested role in disease progression (Fig. 1c and Supplementary Fig. 1K)^15,30,31^. Besides, among the most upregulated genes, we noted *AR*-regulated genes that promote G2–M cell cycle progression, while *AR*-regulated differentiation genes were suppressed, as expected (Fig. 1d)^32–34^.

The progression path indicates that most prostate cancers evolve from normal tissue by continuously increasing *AR* signaling (PC2). Then, under androgen deprivation therapy, the tumors progress to CRPC by increasing cell cycle genes and eventually dedifferentiate to *AR*-negative disease with or without neuroendocrine features (neuroendocrine prostate cancer (NEPC)) (Fig. 1e). Notably, the transcriptional changes correlated well with the protein level changes in an independent set of primary and CRPC samples (Fig. 1f)^35^. Because EZH2 protein quantification was not performed in this dataset, we ascertained its upregulation with disease progression on a tissue microarray (TMA) of 33 primary and matched CRPC samples (Supplementary Fig. 1L)^36^.

Next, we evaluated whether genomic alterations in driver genes correlate with disease progression. We noted a significant correlation of point mutations in *PIK3CA*, *TP53*, *FOXA1*, *KMT2C*, and *PTEN* with progression in primary tumors and *FOXA1* in the metastatic counterpart (Fig. 1g). In primary tumors, we also noticed a positive correlation with *MYC* copy number and an inverse correlation with deletions of *RB1*, *PTEN*, and *TP53*, as expected. In contrast, in CRPC/NEPC samples, only *RB1* loss seemed to correlate well with increased progression (Fig. 1h and Supplementary Fig. 1M, N).

We wondered if pseudotime would also predict survival in patients with metastatic disease. Indeed, increased pseudotime significantly correlated with overall survival (Fig. 1i). While loss-of-function mutations in *RB1* and *TP53* were also associated with poor survival, these alterations did not outcompete pseudotime in the multivariate analysis. Hence, pseudotime still reached significance when only *RB1* wild-type tumors were considered (Supplementary Fig. 1O, P). The data suggest that pseudotime assessment may be useful to predict patient survival in an advanced disease setting.

Finally, we assessed transcriptional changes in key immune pathways throughout tumor progression along the trajectory. It has been widely appreciated during recent years that cancer growth is supported by changes in the tumor microenvironment, such as the polarization of macrophages from an M1— towards M2-like phenotype^37,38^. Indeed, we noticed a potent downregulation of pro-inflammatory M1 markers and an increased and continuous shift towards M2-associated pro-tumorigenic effectors (Fig. 1c, j and Supplementary Fig. 1Q–S). Interestingly, *CD24*—a potent “don’t eat me” signal for M1 macrophages—was associated with progression as well^39^.

### Integration of prostate cancer models in the transcriptome analysis

**Fig. 2:**
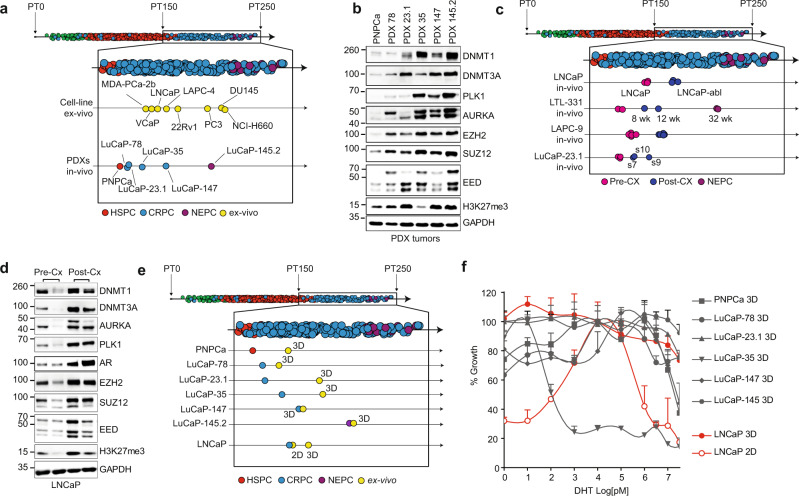
Mapping of human prostate cancer models to the trajectory. **a** Projection of the indicated human cell lines ex vivo and patient-derived xenograft (PDX) models in vivo to the pseudotime (PT) inferred trajectory. Normal prostate: green; hormone-sensitive tumors (HSPC): red; castration-resistant (CRPC) tumors: blue; neuroendocrine (NEPC) tumors: violet; ex vivo cell lines: yellow. Representative replicates for each sample are shown. See Source data file. **b** Immunoblotting analysis of the indicated proteins across PDX models indicates an upregulation of polycomb-repressive complex-2 members and G2M cell cycle checkpoint genes. Experiments were repeated at least three times with similar results. See Source data file. **c** Androgen-dependent xenograft models progress along the trajectory when developing castration resistance. Normal prostate: green; hormone-sensitive tumors (HSPC): red; castration-resistant (CRPC) tumors: blue; neuroendocrine (NEPC) tumors: violet; pre-castration (Pre-CX) PDX models: magenta; post castration (Post-CX) PDX models: dark blue; Wk weeks; s7, s9, and s10 indicate different clones derived from LuCaP-23.1 PDX model. See Source data file. **d** Immunoblot analysis of LNCaP xenograft models shows upregulation of AR, polycomb-repressive complex-2 members, and G2M cell cycle checkpoint genes upon recurrence after castration (Cx). Experiments were repeated at least three times with similar results. See Source data file. **e** Three-dimensional (3D) ex vivo cultures in Matrigel of the indicated PDX and the xenografted LNCaP cells show higher PT than their in vivo counterparts. Normal prostate: green; hormone-sensitive tumors (HSPC): red; castration-resistant (CRPC) tumors: blue; neuroendocrine (NEPC) tumors: violet; ex vivo: yellow. Representative replicates for each sample are shown. Individual replicates are depicted in Supplementary Fig. 2. See Source data file. **f** Dihydrotestosterone (DHT) dose–response curves of the indicated models in 3D using Matrigel versus standard 2D culture. In the 3D conditions, DHT dependency is largely abolished. For each curve, *n* = 3 biologically independent experiments are reported (three animals for each curve). Error bars represent standard errors for each time point. LNCaP: red; PNPCa, LuCaP and PNPCa models: gray. See Source data file.

We next set out to further functionally validate our findings related to disease progression in eight established human prostate cancer cell lines and six PDX models originating either from a surgically, carried off PNPCa^40^ or CRPC (LuCaP-23.1, LuCaP-35, LuCaP-78, LuCaP-145, and LuCaP-147)^41^. To this end, the transcriptional fingerprint of all models clustered towards the outer layer of the progression trajectory (Fig. 2a and Supplementary Fig. 2A, B).

As expected, the PCA positioning of cell lines and the PDX models along the trajectory was highly significantly associated with the originating disease stage and the dependence on androgens (Supplementary Fig. 2C). The hormone-naive (HN) PNPCa model was placed first, followed by the CRPC-derived models, positioned progressively according to their decreasing levels of *AR* dependency. Finally, we observe the AR-negative (PC3, DU-145) and neuroendocrine models (NCI-H660, LuCaP-145.2), which are located at the end of the route (Fig. 2a and Supplementary Fig. 2A, B). As expected, we also noted a corresponding upregulation of key proteins related to polycomb complexes (EZH2, SUZ12 EED), DNA methylation (DNMT1, DNMT3A/B), and G2–M cell cycle progression (Fig. 2b).

Multiple CR sublines of cell lines and PDX models have been generated over the past decades, enabling us to further functionally validate the disease progression trajectory in an isogenic system^42^. Indeed, we found that all sublines progressed on the trajectory (Fig. 2c and Supplementary Fig. 2D–F). Most notably, the LTL-331 PDX model displayed a gradual transcriptional progression from late-stage PNPCa to *AR*-negative, neuroendocrine disease within a timeframe of 32 weeks (Fig. 2c and Supplementary Fig. 2D)^43^. At the molecular level, we also noted an increase in key proteins linked to the trajectory in LNCaP xenograft tumors upon tumor recurrence after castration (Fig. 2d). Altogether, the data suggest that progression along the trajectory can be recapitulated in human cell line and PDX models.

The ex vivo culture of prostate cancer cells has been traditionally a major challenge. That said, the adjustment of the 3D organoid culture system for prostate cancer has enabled the ex vivo culture of PDX-derived cells and the generation of new prostate cancer organoid lines^44,45^. We wondered if the transcriptional output of ex vivo cultures would mirror the corresponding PDX models in vivo. In general, we found that ex vivo organoid cultures displayed a more progressed transcriptional output compared to the corresponding in vivo models (Fig. 2e). In agreement, the *AR* dependency was also largely diminished (Fig. 2f and Supplementary Fig. 2C). This observation could be further validated when androgen-dependent LNCaP cells in standard 2D were cultured in the 3D organoid condition (Fig. 2f). Of note, the standard 2D culture matched better the corresponding xenograft model concerning the position on the progression trajectory (Fig. 2e). In aggregate, the data may suggest that the advances in culturing prostate cancer cells using the organoid system may come at the expense of transformation towards a more progressed and aggressive androgen-independent state.

### Single-cell resolution to the trajectory

**Fig. 3:**
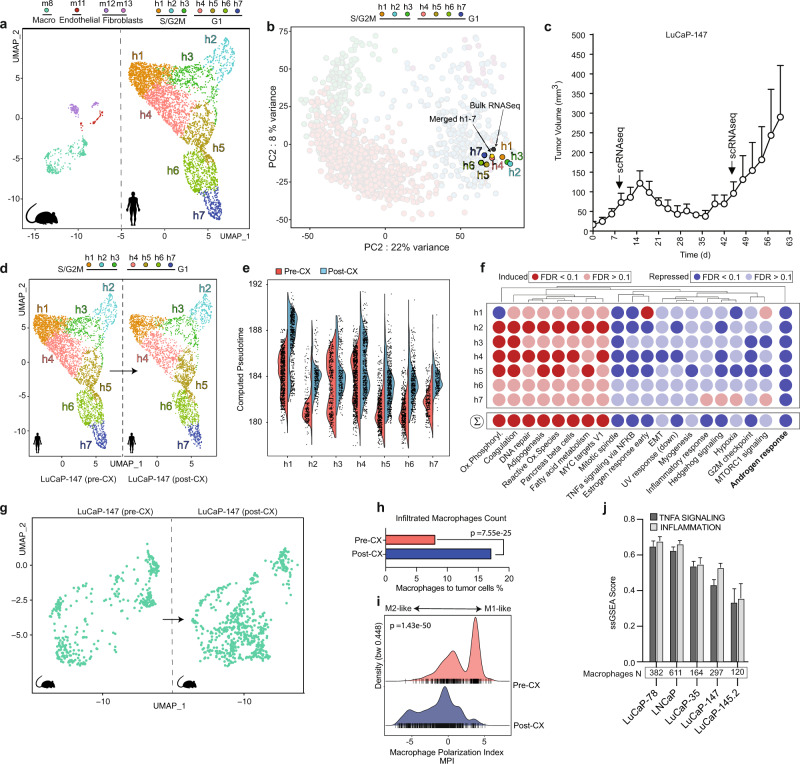
Single-cell resolution to the trajectory. **a** Dimensionality reduction of single-cell distribution of LuCaP-147 PDX model in vivo using Uniform Manifold Approximation and Projection (UMAP) and subsequent identification of cell clusters performed using Seurat^46^ workflow. Human (right) and mouse cells (left) are separated from each other. A total amount of 7 and 4 clusters could be identified for human and mouse cells, respectively. For the latter, we indicated the cells of origin corresponding to the various clusters on top. Inference of cell types was performed with SingleR^86^ through the exploitation of the ImmGen repository^87^. For human cell clusters, we indicated the inferred cell cycle phase as predicted using Seurat. Human and murine cell clusters are depicted using different colors as indicated on top of the figure panel. **b** Projection of single-cell clusters on the PCA plot. The position of merged single-cell data corresponds to the one from bulk RNA-sequencing data. Please refer to the “Methods” section for detailed information on scRNA-seq data integration with bulk RNA-seq. Cell clusters are depicted using different colors as indicated on top of the figure panel. **c** LuCaP-147 xenografts regress and regrow within 4 weeks after castration. *N* = 5 independent experiments. Error bars indicate standard error. **d** Comparison of tumor single-cell clusters before (left) and after castration (right). Cell clusters are depicted using different colors as indicated on top of the figure panel. **e** Violin plot shows an increase in pseudotime of individual cells within the cell clusters after castration. To deal with drop-out events, the pseudotime inference was performed for each cell following imputation of missing genes using RMagic^88^. Pre-castration (Pre-CX): red; post castration (Post-CX): blue. **f** Gene sets perturbed in LuCaP-147 xenografts’ single-cell clusters at regrowth (post castration) compared to pre-castration. Most hallmark gene sets are upregulated (red) or downregulated (blue) similarly. A marked downregulation of *AR*-responsive genes is noted. Differential expression for each cluster denoting the transcriptional changes occurring after castration was determined using the MAST algorithm^91^. Subsequently, we determined the gene-set enrichments using Camera (pre-ranked)^75^. **g** Dimensionality reduction (UMAP) of murine macrophages (green) pre-castration (left) and post castration (right) highlights a notable increase in macrophage count at regrowth. **h** After castration, the percentage of infiltrated macrophage to tumor cell ratio increases. Pre-CX (red): 8.1%; post-CX (regrowth, blue): 17.1%. Statistical significance was computed using Pearson’s *χ*^2^ test. See Source data file. **i** After castration, macrophages display more M2-like transcriptional features according to the macrophage polarization index, as determined by using MacSpectrum^78^. Significance levels (*P* values) were determined using Wilcoxon’s rank-sum text (two-tailed). Pre-CX: red; post-CX (Regrowth): blue. See Source data file. **j** Single samples gene-set enrichment analysis of inflammation-related pathways performed following reclustering of murine macrophages extracted from the corresponding single-cell RNA-seq experiments. Missing gene-expression values (drop-out events) for each cell were imputed using RMagic. With increasing pseudotime along the trajectory, macrophages of xenograft models display less active *TNFA* (dark gray) and inflammatory signaling (light gray). PNPCa xenografts were excluded from the analysis because of the limited number of infiltrated macrophages. Error bars indicate standard error.

We performed single-cell RNA-seq (scRNA-seq) of most aforementioned PDX models in vivo to interrogate the individual cells’ distribution along the trajectory of disease progression. In each case, normal mouse stromal cells were identified and separated from human tumor cells (Fig. 3a and Supplementary Fig. 3A–D). When comparing the merged single-cell data with the previously generated bulk RNA-seq data, we noticed in each case an excellent concordance between the position of both data points on the PCA plot, suggesting that our single-cell data are sufficiently similar to allow the integration into the pan-prostate cancer transcriptome cohort (Fig. 3b and Supplementary Fig. 3E–H).

Subsequently, we interrogated each PDX for the existence of separate subpopulations using the Seurat workflow^46^ (Fig. 3a and Supplementary Fig. 3A–D) and integrated the data into the PCA plot (see “Methods” section). Overall, single cells of the various subpopulations within a given PDX model did not greatly differ in their position to the trajectory and displayed relatively little overlap across PDX models (Fig. 3b and Supplementary Fig. 3E–L). As expected, subpopulations in cell cycle progression (i.e., S and G2M phase) positioned higher on the trajectory (Fig. 3b and Supplementary Fig. 3E–P). That said, the PDX model LuCaP-35 showed a wider distribution of subpopulations along the trajectory with distinct features linked to the S and G2M phase (H1–3 versus H4, 6), respectively, raising the possibility of being composed of two major, biologically diverse tumor clones (Supplementary Fig. 3G, K, O).

Subsequently, we assessed if and how these subpopulations would evolve during progression to androgen independence. For this purpose, we took advantage of the LuCaP-147 PDX tumor model that quickly develops castration resistance and compared the single-cell transcriptional profiles before and after castration (Fig. 3c). Upon regrowth, there was no major difference in the position and abundance of previously identified subpopulations (Fig. 3d). Instead, we noticed a concordant shift along the trajectory for each of the clusters h1–7, which was characterized by a shutdown of canonical *AR* signaling and upregulation of pro-proliferative *MYC* target genes, among others (Fig. 3e, f). Altogether, the data suggest that resistance to castration in this setting occurs likely through reprogramming of the entire tumor cell population instead of a clonal selection of a particular cluster.

Subsequently, we wondered if the induction of resistance may be paralleled by changes in the tumor microenvironment. Indeed, after castration, we observed an increase in the abundance of tumor-associated macrophages that displayed a change in polarization from M1- to M2-like features (Fig. 3g, h). In line with this, we also observed a gradual reduction of *TNFα* signaling and inflammatory signatures—key features of M1 macrophages—in PDX models with increasing pseudotime along the trajectory (Fig. 3i). The results agree with the expression changes of M1- and M2-related transcripts along the trajectory of disease progression described earlier in Fig. 1. Taken together, the data illustrate how bulk transcriptional changes related to disease progression can help to shed light on the emergence of androgen-independent prostate cancer at the single-cell level.

### Co-targeting AR and EZH2 delays tumor progression

**Fig. 4:**
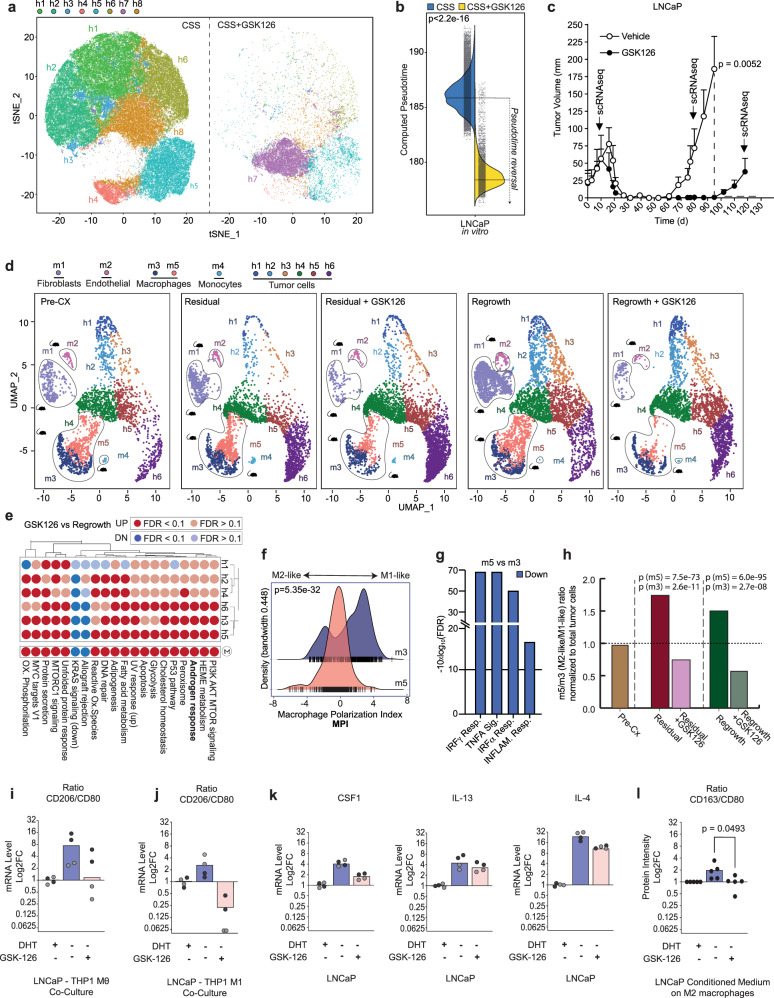
EZH2 inhibition cooperates with castration. **a** Dimensionality reduction (TSNE) of single-cell RNA-seq performed on LNCaP cells cultured in vitro with charcoal-stripped serum (CSS) in the presence (right) or absence (left) of the EZH2 protein inhibitor GSK126. Identification of cell clusters (h1–h8) was performed using the Seurat workflow. EZH2 inhibition has a dramatic impact on LNCaP cells, as most of the clusters disappear, while the remaining cells undergo such deep transcriptional modifications that give rise to a novel cluster (h7). Identified cell clusters are depicted using different colors as indicated on top of the figure panel. **b** Pseudotime of individual LNCaP cultured in charcoal-stripped serum (CSS, blue) is significantly reduced upon GSK126 (CSS + GSK126, yellow) treatment. Pseudotime was computed for each cell, following imputation of missing genes (dropouts) using RMagic. Statistical significance was determined using Wilcoxon’s test. See Data source file. **c** GSK126 treatment for 3 weeks upon castration significantly delays the regrowth of LNCaP xenografts after castration. Curves are determined from *n* = 6 animals per group. Statistical significance at 100 days was asses using an unpaired, two-tailed Student’s *t* test. **d** Dimensionality reduction (UMAP) of LNCaP xenografts performed on scRNA-seq experiments derived from mice before castration (Pre-CX, left), 80 days after castration (residual/post-CX, left-center), 80 days after concomitant castration and EZH2 inhibition with GSK126 (residual/post-CX + GSK126, center), 120 days after castration (regrowth/post-CX, right-center), and 120 days after concomitant castration and EZH2 inhibition with GSK126 (regrowth/post-CX + GSK126, right). Experiments were performed by sequencing one mouse per condition. Murine cells can be subdivided into five clusters corresponding to different cell populations according to SingleR (m1: fibroblasts; m2: endothelial cells; m3, m5: macrophages; m4: monocytes). Human malignant cells can be separated into six clusters. An increase in the relative number of cells in cluster h6 and a concomitant reduction of murine macrophages following EZH2 inhibition is observed. Identified human and murine cell clusters are depicted using different colors as indicated on top of the figure panel. **e** Upon GSK126 pretreatment, for each cluster, we determined differentially expressed genes (MAST algorithm) and performed gene-set enrichment using Camera (pre-ranked). Results highlight a global increase in androgen-responsive genes. Red: upregulated; blue: downregulated. See Source data file. **f** The density plot of macrophage polarization index (MPI) reveals that macrophage cluster m5 (which decreases upon GSK126 administration) shows M2-like transcriptional features, while cluster m3 corresponds to an increased M1-like polarization as determined by MacSpectrum. Statistical significance was determined using Wilcoxon’s test. Blue: density distribution of m3 cells; red: density distribution of m5 cells. See Data source file. **g** Differential expression (MAST algorithm) shows that M1-like inflammatory signaling pathways are downregulated in m5 compared to the m3 cluster. Blue: downregulated. **h** Histogram representing the relative ratio between m5- and m3 cluster before castration (pre-CX, brown), 80 days after castration (residual, red) and 120 days after castration (regrowth, green). Macrophage population belonging to m5 cluster (M2-like) decreases upon treatment with GSK both at 80 (residual + GSK, pink) and 120 days (regrowth + GSK, light green). The decrease in m3 cluster is much less pronounced. *P* values for both m3, and m5 subpopulations were determined using *χ*^2^ test and adjusted for multiple comparison using Bonferroni’s correction method. **i**–**j** Treatment with GSK126 inhibits the M2 polarization of THP1-Mθ and THP1-M1 macrophages. **i** Bar graph showing the log_2_ fold change ratio of M2 versus M1 surface markers, in THP1-Mθ (*n* = 2 independent experiments) or THP1-M1 (**j**) macrophages cocultured for 72 h with LNCaP maintained in charcoal-stripped serum (CSS) alone or supplemented with 1 nM DHT or 1 μM GSK126 (*n* = 2 independent experiments). Technical replicates are indicated using dots of different colors. Experment 1: black; Experiment 2: gray. The surface expression of the CD80 (M1) and CD206 (M2) surface markers was determined by flow cytometry. The mean fluorescence intensity of the positive cells was compared with the average signaling in the DHT condition to calculate the fold change. The ratio was determined by comparing the fold change of the M2 surface marker versus the M1 surface marker. CSS: blue; CSS + DHT: gray; CSS + GSK126: pink. See Source data file. **k** Bar graph showing the gene-expression changing in LNCaP cells of cytokine associated with M2 polarization. Gene-expression levels were measured by RT-qPCR into LNCaP maintained for 4 weeks in DHT, CSS, and GSK126. The log_2_ fold change was calculated using actin as reference genes and compared to the DHT condition (*n* = 2 independent experiments per condition). Technical replicates are indicated using dots of different colors. Experment 1: black; Experiment 2: gray. CSS: blue; CSS + DHT: gray; CSS + GSK126: pink. See Source data file. **l** Treatment with GSK126 reverts the polarization of human M2-like macrophages. Bar graph showing the log_2_ fold change ratio of M2 (CD163) versus M1 (CD80) surface markers and human M2-like macrophages polarized for 7 days using the supernatant of LNCaP cells maintained for 4 weeks in DHT, CSS, and GSK126, respectively (*n* = 5 independent experiment). CSS: blue; CSS + DHT: gray; CSS + GSK126: pink. See Source data file. Significance was computed using Student’s *t* test, two-tailed, paired. No *P* value correction was applied as only one comparison was performed.

Because *EZH2* emerged as a top upregulated transcript within the trajectory of disease progression and had been shown to promote androgen independence^15,30,47,48^, we set out to investigate if co-targeting *AR* and *EZH2* may prevent or substantially delay disease progression. Indeed, we noted a dramatic change in the transcriptional output program of LNCaP cells when treated with the EZH2 protein inhibitor GSK126 under androgen-deprived culture conditions in charcoal-stripped serum (CSS) (Fig. 4a). Previously detected LNCaP subpopulations (h1–6, h8) formed a new subpopulation (h7), suggesting a nearly complete rewiring of transcription, upregulation of *AR* signaling, reduction of E2F-related cell cycle genes, and reversion of progression on the trajectory (Fig. 4b and Supplementary Fig. 4A–D). In line with this, we noticed a strong reduction in colony formation when androgen-dependent LNCaP, VCaP, and LAPC4 cells were subjected to CCS and treated with GSK126, while forced expression of *EZH2* was sufficient to promote colony formation in the same setting (Supplementary Fig. 4E).

Next, we tested if our observations would also translate into an in vivo setting. For this purpose, we injected LNCaP cells into the flank of immune-compromised mice and treated the emerging xenograft tumors with castration alone or in combination with 3 weeks of GSK126. In both cases, the tumors fully regressed. While the tumors of castrated mice regrew with a latency of around 4 weeks, GSK126 co-treated tumors took more than twice as much time to re-initiate tumor growth (Fig. 4c).

We subsequently performed scRNA-seq on the tumors pre- and post castration to investigate transcriptional changes on tumor and stromal cell subpopulations. As noted previously for LuCaP-147, we found no major change in the tumor cell subpopulations (i.e., h1–6) that adapted to castration (Fig. 4d). Because GSK126 treatment in vivo had been stopped for 3 months before harvesting the tumors, the transcriptional changes in the tumor cells appeared less striking than in the aforementioned cell culture setting (Fig. 4a, d). That said, we observed after GSK126 co-treatment a continuous relative increase in tumor cell numbers of cluster h6—the least progressed cluster on the trajectory that also displayed the highest *AR* mRNA levels (Fig. 4d and Supplementary Fig. 4F–I). This cluster showed a further increase in *AR* signaling and a reversion of disease progression after GSK126 treatment (Fig. 4e and Supplementary Fig. 1). Finally, we assessed if pharmacologic inhibition of *EZH2* may also affect the tumor microenvironment. Indeed, GSK126 treatment suppressed the continuous proliferation of fibroblasts after castration and tumor regrowth (Supplementary Fig. 5A, B). In line with this, GSK126-treated LNCaP cells suppressed the secretion of the fibroblast growth factors PDGF-AA and PDGF-AB-BB in culture^49^ (cytokine arrays, Supplementary Fig. 5C, D) Importantly, xenograft-associated macrophages also continuously increased in numbers and displayed a shift towards M2-like polarization in tumors adapted to castration as previously observed (Fig. 4d, f, g). Strikingly, we found a pronounced relative reduction of preferentially M2-like macrophages in GSK126-pretreated tumors, suggesting that GSK126-mediated changes on the tumor microenvironment may have contributed as well to the delayed regrowth of LNCaP xenografts (Fig. 4h).

We further assessed the interaction between LNCaP cells and macrophage-like THP1 cells in vitro. The supernatant from THP1/LNCaP cocultures in CSS promoted M2 polarization on M0- and M1-THP1 cells, respectively (Fig. 4i, j and Supplementary Fig. S5E–I). At the same time, GSK126 reverted M2 polarization (Fig. 4i, j). In line with this, GSK126 suppressed the induction of cytokines capable of M2 induction in LNCaP cells (i.e., *CSF1*, *IL13*, *IL4*; Fig. 4k and Supplementary Fig. S5J). Indeed, the supernatant of GSK-treated LNCaP cells was sufficient to revert CSS-induced polarization of human M2 macrophages (Fig. 4l and Supplementary Fig. S5K, L).

In aggregate, the data suggest a rationale for joint targeting of *AR* and *EZH2* in prostate cancer because the latter reverts tumor cell progression towards a more androgen-dependent state and at the same time counteracts adaptive changes in macrophages and fibroblasts that are intimately linked to disease progression.

## Discussion

In the present study, we combine transcriptional profiles of prostate cancers at various disease stages to a comprehensive Prostate Cancer Transcriptome Atlas with negligible study-related interference (i.e., batch effects). Mining the atlas reveals a rather uniform trajectory towards disease progression from normal prostate, primary, and metastatic CRPC. The trajectory is characterized by a gradual upregulation of genes related to *EZH2*-mediated polycomb signaling and cell cycle progression, most namely G2M checkpoints and mitotic spindle genes. The latter may provide an explanation why taxanes (i.e., docetaxel, cabazitaxel), which disrupt microtubule function during cell division, remain a cornerstone of prostate cancer treatment in the hormone-sensitive and CR metastatic setting^50–57^.

*EZH2* has been previously described to be critically involved in prostate cancer as an activator of *AR* signaling^30^. It is also a key component of PRC2-mediated gene silencing—a developmental pathway implicated in dedifferentiation and prostate cancer progression^15,29,58–66^. In agreement with the latter, we find *EZH2* the top upregulated gene in the progression trajectory along with other PRC2 members. In line with a function in driving disease progression and dedifferentiation towards the loss of *AR* expression, we demonstrate how *EZH2* inhibition reverts the transcriptional output of prostate cancer cells along the progression trajectory. The findings may have important implications for the treatment of prostate cancer patients in an HN or early CRPC because it may prevent the dedifferentiation of cancer cells as an escape mechanism to *AR*-directed therapeutic interventions. In line with previous reports, we noticed along the trajectory a change of macrophage polarization from inflammatory M1 to pro-tumorigenic M2^37,38^. Our findings further underscore the antitumor potential of pharmacologically re-educating macrophages towards M1. Castration was sufficient in our PDX models to induce a change toward M2 polarization after a relatively short period in line with previous reports^67^, suggesting that therapeutic interventions per se may be at least in part the underlying cause. Importantly, we show in the same setting that inhibition of EZH2 protein substantially blocked the castration-induced polarization change towards M2, uncovering a thus far underappreciated role for *EZH2* in macrophage polarization another rationale towards co-targeting *AR* and *EZH2* in prostate cancer.

It is mostly unknown how disease progression in prostate cancer emerges at the single-cell level. Using a series of PDX models reflecting different progression stages from HN to *AR*-negative late-stage disease enabled the addition of single-cell resolution to the progression trajectory. Our results suggest that resistance to androgen deprivation may occur through transcriptional adaptation of tumor cells towards a more progressed state. In line with this, a recent study has proposed that prostate regeneration (a process that shares many molecular features with prostate cancer progression) is driven by nearly all persisting luminal cells, not just by rare stem cells^68^. That said, in our study, we have used a relatively uniform xenograft tumor model that has been already derived from CRPC and thus adapt swiftly to castration in mice. Conceivably, resistance to androgen receptor inhibition over a longer period may also involve the selection of stem-cell-like subpopulations irrespective of the presence of genetic drivers of CRPC (e.g., *AR* amplification or point mutations)^69–73^.

We provide a web-based interface for the research community to facilitate the mining of the Prostate Cancer Transcriptome Atlas, called the PCaProfiler (https://www.prostatecanceratlas.org/). Using this resource, we readily identify, for example, that a subpopulation of very advanced prostate cancer tissues expresses high levels of *IL23A*, a cytokine recently described to mediate castration resistance in prostate cancer^74^. Interestingly, correlating the *IL23A* expression with genomic features in our webtool identifies a tight association of *IL23A* expression with gains and amplification of its receptor *IL23R*. Such insights may be important for patient selection/stratification for anti-*IL23* targeting monoclonal antibodies under clinical development (i.e., NCT04458311).

The PCaProfiler will also allow the pseudotime annotation of new cancer transcriptomes. In a clinical trial setting, this information may enable identifying antitumor responses within a certain subset of patients with a given degree of disease progression. In a preclinical setting, the atlas may also help researchers to choose the corresponding model system that reflects the disease stage under investigation. Of note, in this regard, we have already annotated the pseudotime for the most frequently used prostate cancer cell lines (see PCaProfiler). Alternatively, the PCaProfiler may enable researchers to verify and optimize the ex vivo culture condition so that it best mirrors the in vivo setting.

In conclusion, we successfully merged the RNA-seq data from several prostate cancer studies, covering different disease stages. Based on that, we delineate the roadmap to prostate cancer progression in a qualitative and a quantitative manner. Furthermore, we also show how individual tumor cells can be tracked along the progression trajectory in response to pharmacological perturbations. Because the transcriptome data of advanced metastatic disease will become more readily available for other tumor types, the current study may serve as a blueprint for their analysis and exploitation.

## Methods

### Experimental model and subject details

#### Plasmids

The pHAGE-puro (Plasmid #118692) and the pHAGE-EZH2 (Plasmid #116738) were purchased from Addgene.

#### Cell lines

PC3, DU-145, 22rV1, MDA-PCa-2b, LAPC4, LNCaP, VCaP, and HEK 293T cell lines were purchased from ATCC (American Tissue Culture Collection) (Manassas, USA). The LAPC4 cell line was a gift from Prof. Helmut Klocker, the LNCaP-abl cell line was a gift from Prof. Myles Brown (DFCI, Boston), and the THP1 cell line was a gift from Prof. Saverio Minucci (IEO, Milan). The supernatant of all cell lines was routinely tested (two times per month) using the MycoAlertTM Mycoplasma Detection Kit (Catalog #: LT07-318, Lonza). All cell lines resulted negative for Mycoplasma infection.

### Immunohistochemically staining

EZH2 protein abundance was analyzed on TMA, including matched primary and CRPC samples^36^ (University of Bern). All prostate cancer samples from human subjects were obtained under approval by the Ethics Committee of Northwestern and Central Switzerland (EKNZ, Nos. EK/1311 and 2015/228). This is based on a retrospective study. Tumor‐free prostate core needle biopsies were used to analyze benign prostate (*n* = 3 patients). Prostate cancer biopsies included in the TMA were taken during routine clinical treatment. Samples were selected based on the following inclusion criteria: (a) histologically diagnosed PCa, (b) tumor‐containing biopsies available at HN and CR state, and (c) sufficient quality and amount of material, as evaluated by experienced pathologists (JPT). Castration resistance was defined as either biochemical progression (i.e., serum PSA progression according to the *Prostate Cancer Clinical Trials Working Group* criteria or clinical progression. A TMA comprising 112 matched HN/CR tissue specimens and including 107 transurethral resections and five distant metastases derived from 55 PCa patients was constructed. Briefly, tissue cylinders with a diameter of 1 mm were punched from the patient’s tissue blocks containing the specimens using the robotic precision instrument Grand Master TMA (3D Hitech). Tissue cylinders were placed in one recipient paraffin block. After the block construction was completed, an 8 μm section of the resulting TMA block was cut to a microtome. Due to tissue loss, a common problem associated with TMA technology, 33 high-quality matched tissue samples of primary and CRPC remained after sectioning.

For EZH2 IHC, slides were analyzed with the Bond-III Automated Staining System (Leica) using manufactured reagents for the entire procedure. For antigen retrieval, slides were incubated for 60 min in citrate buffer at pH 6 at 98 °C. Thereafter, slides were incubated with a rabbit monoclonal antibody against EZH2 (D2C9, CST5246 from Cell Signaling) at the dilution of 1:500. Detections were performed using the detection refine DAB Kit (Leica). Immunohistochemical staining was evaluated as the percentage of tumor cells with nuclear positivity for EZH2 using Aperio ImageScope (Leica).

### Cell culture

PC3, DU-145, 22rV1, LAPC4, LNCaP, and THP1 cell lines were cultured in RPMI-1640 (21875-034, Life Technologies) supplemented with 10% fetal bovine serum (FBS-11A, Capricorn Scientific), and 1% penicillin/streptomycin (15140-122, Life Technologies) with 5% CO_2_ at 37 °C. LAPC4 was also supplemented with 1 nM dihydrotestosterone (DHT).

The LNCaP-abl cell line was cultured in phenol red-free RPMI-1640 (11835063, Life Technologies) containing 10% CSS (FBS, charcoal-stripped, A3382101, Life Technologies) and 1% penicillin/streptomycin with 5% CO_2_ at 37 °C.

VCaP and HEK 293T cell lines were cultured in Dulbecco’s modified Eagle’s medium (DMEM) (61965059, Life Technologies) supplemented with 10% FBS and 1% penicillin/streptomycin with 5% CO_2_ at 37 °C.

MDA-PCa-2b cell line was cultured in ATCC-formulated F-12K medium (30-2004) supplemented with 20% FBS, 25 ng/ml cholera toxin (C8252, Sigma), 10 ng/ml epidermal growth factor (EGF) (AF-100-15, PeproTech), 0.005 mM phosphoethanolamine (P1348, Sigma), 100 pg/ml hydrocortisone (H0135, Sigma), 45 nM selenium acid (211176, Sigma), 0.005 mg/ml human recombinant insulin (I1884, Sigma), and 1% penicillin/streptomycin with 5% CO_2_ at 37 °C.

### PEI-mediated transfection and lentiviral infection

Lentiviral production was carried out by polyethylenimine (PEI)-mediated transfection of the HEK 293T with PHAGE (Empty, Addgene 118692) and PHAGE-EZH2 (Addgene 116738) vectors. Briefly, The HEK 293T cells were seeded in a 10 cm culture dish (4 × 10^–6^ cells/plate) and incubated overnight at 37 °C in a 5% CO_2_ humidified atmosphere. After 24 h, the vector plasmid (PHAGE or PHAGE-EZH2; 3 μg), the packaging plasmid (pCMV-dR8.2; 2.7 μg), and the envelope plasmid (pVSV-G; 0.7 μg) were mixed in Opti-MEM™ I Reduced Serum Medium (300 μl/10 cm culture dish, Thermo Fisher 31985070) with 1.25 mM PEI (Sigma-Aldrich, 919012) solution (ratio μl PEI:μg DNA 4:1). The DNA/PEI mixtures were incubated at room temperature for 15 min. The DNA/PEI mixture was then added to the supernatant of the HEK 293T cell. Forty-eight hours after the transfection, the viral supernatants were collected and filtered through a 0.45 m filter. The LNCaP cell line was incubated with viral supernatant and 8 μg/ml Polybrene (H9268, Sigma) for 72 h and then selected with 2 μg/ml puromycin (P8833, Sigma) for 2 weeks. Western blotting was used to verify EZH2 protein abundance.

### Animal experiments

All animal experiments were carried out accordingly to protocol approved by the Swiss Veterinary Authority/Board (TI-42-2018 and TI-10-2010) and received approval by the ethical committee of the Institute of Oncology Research. All in vivo studies used 6–8-week-old male NRG (NOD-*Rag1*^*null*^ *IL2rg*^*null*^, NOD rag gamma) mice.

### Housing conditions

Before experimental procedures, mice were housed in individually vented cages, maintained at room temperature (20–22 °C) and a 12 h daylight cycle. Groups of five mice were kept in individual cages of ~465 cm^2^. The cages were sealed, autoclaved before use, and used in a “Sealsafe” rack (Techniplast) with a 0.2-μm aerosol bacteria barrier vent. AII manipulation of the cages (e.g., to replace bedding) occurred in a cage changing station (CCS, Techniplast), designed to maintain animals in a sterile airflow environment. For experimental procedures, mice were housed in groups of 4–5 mice in ~355 cm^2^ filter-topped cages, on racks in a specified pathogen-free barrier facility. Cages and filters were autoclaved before use, and experimental procedures and manipulation of the cages occurred in a sterile laminar flow hood (Skan AG). PDX LuCaP-147, LuCaP-145.2, LuCaP-78, LuCaP-35, and LuCaP-23.1 were provided by Dr. Eva Corey^41^. The LuCaP PDX series has been established by subcutaneous transplantation of tumor tissue of patients with metastatic prostate cancer tumors, from 1991 to 2005. Tissue collection for research was approved by the University of Washington Human Subjects Division IRB, which approved all informed consents that were used for tissue acquisition (IRB #39053). Dr. Marianna Kruithof-de Julio provided PNPCa. The established PDX was originated from a patient who presented with primary PCa (Gleason 9). Orchiectomy was performed directly after biopsy sampling; thus, the tumor was androgen-dependent at the time of collection. The patient included in the study provided written informed consent (Cantonal Ethical approval KEK 06/03 and 2017-02295). PDXs tumors were maintained by subcutaneous implantation of Matrigel-embedded tumor fragment (1–2 mm average diameter tumor or take rate varied from 1 to 6 months). For the experiment in castration LNCaP or LuCaP-147 cells (obtained from tumor dissociation, see details in the section “ex vivo culture of PDXs”) were suspended in PBS and 50% Matrigel and subcutaneously injected into the dorsal flanks of the mice (2 × 10^6^ cells/mouse). Tumor growth was recorded using a digital caliper, and tumor volumes were calculated using the formula (*L* x *W*^2^)/2, where *L* is the length and *W* the width of the tumor. Tumor volume was measured two times per week. When the tumor reached the dimension of 50–100 mm^2^, mice were surgically castrated. For the GSK126 treatment, the mice were treated 1 week after castration by daily intraperitoneal injection at a dose of 100 mg/kg for 3 weeks. At the end of the experiment, mice were euthanized, tumors explanted, and used for molecular assessment.

### Ex vivo culture of PDXs

PDX tumor tissue was cut into small pieces (1–0.5 mm) with a scalpel blade and then digested in Collagenase Type I media solution (200 U/ml, Catalog #: SCR103, Millipore) at 37 °C for 45–60 min. After enzymatic dissociation, the cell suspension was passed through a 100 µM cell strainer (11814389001, Roche) to eliminate macroscopic tissue pieces and centrifuged. The cell pellet was then resuspended in 2-volume RBC lysis buffer (11814389001, Roche), incubated for 3 min at room temperature (RT), and, after centrifugation, resuspended in a complete media that permitted the propagation of PDX cells by 3D culture. The completed medium consisted of advance DMEM/F12 (Thermo Fisher, 12634010) supplemented with 2 mM glutamine (Thermo Fisher, 25030032), 1% HEPES (Thermo Fisher, 15630080), 1% B27 supplement (Life Technologies, 17504-044), 10 mM nicotinamide (Sigma-Aldrich, N0636), 5 ng/ml EGF (PeproTech, AF-100-15), 5 ng/ml fibroblast growth factor-2 (FGF-2) (PeproTech, 100-18B), 10 ng/ml FGF-10 (PeproTech, 100-26), 100 ng/ml Nogging (PeproTech, 120–10 °C), 1 μM prostaglandin E (Bio-Techne, AG 2296), 500 nM A83-01 (Bio-Techne, AG 2939), and 1% of R1-spondin-1 (conditional medium of R-spondin-1-expressing 293T cell line). The single-cell suspensions in complete medium (density 10^6^ cells/ml) were finally embedded in 50% phenol red-free Matrigel (356231, Corning) and plated as a drop in a 96-well-plate (10,000 cells/well in a single drop of 10 μl Matrigel) and maintained in the medium for 7–10 days.

### DHT dose–response assay

3D culture of PDXs (see section “Ex vivo culture of PDXs”) or 2D culture of LNCaP cell line (5000 cells/well in 10% CSS medium) were seeded in triplicate in a 96-well plate and subsequently treated with serial dilutions of DHT (concentration range of 0.01 nM–30 µM). Proliferation was assessed after 7–10 days by CellTitre-Glo assay (G9241, Promega) for 3D culture or MTT (methylthiazolyldiphenyl-tetrazolium bromide) assay (M5655, Sigma) for the 2D culture. For each time point, absorbance (OD, 590 nm) was measured in a microplate reader (Cytation 3 Imaging Reader Biotek).

### Colony formation assay in DHT-free medium

VCAP (5 × 10^5^ cells/well), LAPC4 (2.5 × 10^5^ cell/wells), LNCaP (2.5 × 10^5^ cells/well), or LNCaP-overexpressing EZH2 were seeded in triplicate in 6-well plates in a standard medium. After 24–48 h, when the cells were attached to the plate and formed a confluent layer, the medium was replaced with 10% CSS medium (DHT-free medium) with/without 1 µM GSK126 and kept in culture until the formation of the colonies (4–6 weeks). The medium/treatment was weekly replaced. At the end time point, the cells were gently washed with PBS, fixed with 0.01% crystal violet, and 20% of EtOH for 30 min, and then wash out with water. The images of colonies were acquired using the Fusion Solo IV LBR system and the quantification of colonies was performed by the ImageJ software.

### Antibodies and Western Blot Analysis

The primary antibodies used were: anti-GAPDH (sc-47724, Santa Cruz), anti-AR (sc-7305, Santa Cruz), anti-DNMT3A (sc-365769, Santa Cruz), anti-EZH2 (612667, BD Transduction Laboratory), anti-DNMT(5032S, Cell Signaling Technologies), anti-EED (85322S, Cell Signaling Technologies), anti-SUZ12 (3737S, Cell Signaling Technologies), anti-Aurora A (14475T, Cell Signaling Technologies), antiH3K27me3 (9733S, Cell Signaling Technologies), and anti-PLK1 (B290751, BioLegend).

Tumor tissues (25–30 mg) or cellular pellets were lysates with RIPA buffer supplemented with cocktail phosphatase inhibitors (4906845001, Roche) and proteases inhibitors (5892953001, Roche). Protein concentration was determined by BCA reagent (A52255, Thermo Fisher Scientific); 30–50 μg of whole protein lysate was separated on 8–12% SDS–polyacrylamide gels and transferred onto PVDF membrane (88518, Thermo Fisher Scientific). The membranes were blocked with 5% milk in Tris-buffered saline with Tween-20 (TBST) for 30 min at RT, incubated overnight at 4 °C with primary antibodies, and incubated for 1 h at RT with secondary antibodies (anti-rabbit IgG HRP W401B and anti-mouse IgG HRP, W402B, Promega). The protein bands were visualized using the western bright quantum reagent (K-12042-D20, Advansta) and quantified using the Fusion Solo IV LBR system.

### Cytokine array profile

Profile of cytokines, chemokines, and other proteic soluble factors contained in LNCaP-conditioned medium (CM) was detected by using Human XL Cytokine Array Kit (ARY022B, R&D System) as reported in a manufacturer’s protocols. LNCaP cells were maintained for 4 weeks in CSS alone or CSS supplemented with 1 nM DHT or with 1 µM GSK126. The medium was changed two times for a week. Five hundred microliters of each cell culture supernatant was run on each array for 24 h. Pixel density plots were detected The protein bands were visualized using the chemiluminescent detection reagent included in the kit, and quantified using the Fusion Solo IV LBR system. The images were analyzed using the Image Lab software. The signal (pixel density) of each duplicate spot was determined. The signaling of a negative spot control was used as a background value. The average background signal was subtracted from each spot. The signaling of each spot was normalized using the average signaling of two different array-specific positive controls. To calculate the average fold changes (FCs), the signaling of each normalized spot was compared with the average normalized signal of the corresponding DHT-treated condition.

### RT-qPCR analysis

According to the manufacturer’s guidelines, the RNA extraction was performed from a cellular pellet of THP1-derived macrophages using an RNeasy Kit (74106, Qiagen). Quantitative reverse transcription PCR (RT-qPCR) was carried out using KAPA SYBR® FAST One-Step (KK4600, Sigma) following the manufacturer’s protocol. The primer sequences were obtained from PrimerBank (http://pga.mgh.harvard.edu/primerbank/index.html). The list of the primer is reported in Supplementary Data 3. Actin was used as a housekeeping gene. The qPCR analysis was performed using the 2^−ΔΔCt^ method.

### Flow cytometry analysis

For phenotype analysis, isolated THP1-derived macrophages or human M2-like macrophages were suspended in PBS containing 1% fetal calf serum and then stained for 30–45 min at RT with a cocktail of surface marker antibodies. For staining anti-CD14-BV650, anti-CD80-PE, anti-CD163-PECy7, and anti-CD206-BV510 antibodies (eBioscience) were used. Samples were acquired on a BD LSR-Fortessa flow cytometry (BD Biosciences). Data were analyzed using the FlowJo software. FACS gating/sorting strategy is indicated in Supplementary Fig. 6.

### In vitro differentiation of THP1-derived macrophages

The THP1 cells were polarized to M1-like macrophages in RPMI medium,10% FBS, and 100 ng/ml of phorbol‑12 myristate‑13 acetate (PMA) (Sigma, P1585) for 24 h following stimulation with 10 ng/ml interferon-γ (300-02, PeproTech) and 10 ng/ml of lipopolysaccharide (Sigma, 916374) for 48 h. The unpolarized THP1-Mθ were derived from THP1 cells stimulated for 48 h only within PMA in RPMI medium and 10% FBS. After stimulation, the TH1-M0 or the THP1-M1 cells were maintained in CSS, CSS 1 nM DHT, or CSS 1 µM GSK126 fresh medium or cocultured with LNCAP cells.

### In vitro differentiation of peripheral blood mononuclear cell-derived macrophages

Buffy coat was mixed 1:2 with PBS. The mixture was then added 3:1 to Ficoll gradient (Invitrogen, 17-1440-03) and spun down at 1500 r.p.m. for 25 min at RT (w/o brakes). The leukocyte ring was collected and washed with PBS. Cells were then resuspended in 0.5% bovine serum albumin in PBS containing anti-CD14 microbeads (130-050-021, Milthenyi Biotec) and purified for positive selection using LS MACS column system (130-042-401, Miltenyi Biotec). The purity of CD14^+^ cells was analyzed by FACS using an anti-CD14-BV650 antibody (eBioscience). The CD14^+^ cells were polarized to M2-like macrophages in RPMI medium, 10% FBS, 50 ng/ml granulocyte-macrophage colony-stimulating factor (R&D Systems, 215-GMP), 50 ng/ml macrophage colony-stimulating factor (R&D Systems, 216-GMP), 20 ng/ml interleukin-13 (200-13, PeproTech), and 20 ng/ml of IL4 (200-04, PeproTech) for 7 days. In the experiments with CSS, CSS 1 nM DHT, or CSS 1 µM GSK126 fresh medium and LNCaP-CM, the CD14^+^ cells were polarized to M2-like macrophages using the same cocktail of cytokine.

### RNA extraction for RNA-seq analysis

According to the manufacturer’s guidelines, the RNA extraction was performed from PDX’s frozen fragment (25–30 mg) of cellular pellet using RNeasy Kit (74106, Qiagen). The RNAs were processed using the NEB Next Ultra II Directional Library Prep Kit for Illumina (E7765, NEB) and sequenced on the Illumina NextSeq500 with single-end, 75-base-pair-long reads.

### Single-cell isolation for scRNA-seq

To perform scRNA-seq PDX tumor tissue, they were dissociated into single cells as described above (see section “Ex vivo culture of PDX”). After resuspension in PBS, single-cell suspensions were loaded into a 10x Chromium Controller (10x Genomics, Pleasanton, CA, USA), aiming for 10,000–5000 cells, with the Chromium Next GEM Single Cell 3′ v3.1 Reagent Kit (PN-1000121, 10x Genomics), according to the manufacture’s instructions.

### RNA-seq data processing

#### Sequencing of xenografts and 2D and 3D cultures

We retrieved bulk RNA-seq data for cellular models of prostate cancer from various available datasets and extended these by performing bulk RNA-seq of several prostate cancer Xenografts models (i.e., PNPCa; LuCaP-78, LuCaP-23, LuCaP-35, LuCaP-145; LNCAP), and their derived 3D cultures. Additional sequencing was performed for 2D cultures of LNCaP, LNCaP-all, LAPC4, and VCaP cells (see “Data availability” section).

#### Prostate Cancer Transcriptome Atlas

To build an integrated resource of transcriptional features representing all stages of prostate cancer progression, we collected raw sequencing data from a large panel of independent datasets. We gathered raw data for 1223 clinical samples (1104 excluding technical replicates, 1044 excluding multiple metastatic sites derived from the same individual). The resulting integrated cohort is representative of various stages of disease progression, namely, normal prostate specimens (*n* = 174), primary tumors (*n* = 714), CRPCs (*n* = 316), and CRPCs showing features of neuroendocrine trans-differentiation (*n* = 19). Raw sequencing files were retrieved from following sources: (1) Gene Tissue Expression Database; (2) The Cancer Genome Atlas (TCGA); (3) atlas of RNA-sequencing profiles of normal human tissues (GSE120795); (4) integrative epigenetic taxonomy of PNPCa (GSE120741); (5) prognostic markers in locally advanced lymph node-negative prostate cancer (PRJNA477449); (6) the long noncoding RNA landscape of NEPC and its clinical implications (PRJEB21092); (7) integrative clinical sequencing analysis of metastatic CRPC reveals a high frequency of clinical actionability (PRJNA283922; dbGaP: phs000915); (8) CSER—exploring precision cancer medicine for sarcoma and rare cancers (PRJNA223419; dbGaP: phs000673); (9) molecular basis of NEPC (PRJNA282856; dbGaP: phs000909); (10) heterogeneity of androgen receptor splice variant-7 (AR-V7) protein expression and response to therapy in CRPC (GSE118435); (11) molecular profiling stratifies diverse phenotypes of treatment-refractory metastatic CRPC (PRJNA520923; GEO: GSE126078). Depending on the specific dataset considered, *fastq* files were downloaded either by using gdc-client (TCGA) or sra-toolkit (SRA, dbGaP). Detailed information along with all available clinical annotations are provided in Supplementary Data 1.

#### RNA-seq data processing of clinical samples

The overall quality of sequencing reads was evaluated using FastQC (v.0.11.9). Sequence alignments to the reference human genome (GRCh38) were performed using STAR (v.2.6.1c) in two-pass mode, to significantly increase sensitivity to novel splice junctions compared to the regular single mapping. Briefly, in the two-pass mapping procedure, reads are mapped twice: in the first pass, the novel junctions are detected and inserted into the genome indices; in the second pass, all reads are re-mapped using annotated (from the GTF file) and novel (detected in the first pass) junctions. In particular, gene expression was quantified at the gene level in the second pass by using the comprehensive annotations made available by Gencode (v29 GTF File). Strand-specific information was not maintained to avoid technical differences between stranded and unstranded libraries. Samples were adjusted for library size and normalized with the variance stabilizing transformation (vst) in the R statistical environment using DESeq2 (v1.28.1) pipeline. When performing differential expression analysis between groups, we applied the embedded *IndependentFiltering* procedure to exclude genes that were not expressed at appreciable levels in most of the samples considered. If not otherwise specified, all GSEAs were performed using the limma (v.3.46.0) package (Camera, use. ranks set to TRUE)^75^. Gene-set collections were retrieved either from the Molecular Signature Database (MSigDB) or from previous publications (AR/NE-Score)^76^. *P* values were corrected for multiple testing using the false discovery rate (FDR) procedure, with the significance threshold set to 0.05. In addition, GSEA significance was logarithmically transformed in form of −10log_10_(*p*-adjusted), with a bold intercept (*x* = 13.01) indicating the FDR threshold depicted in the corresponding plots.

#### Batch-effect correction and PCA

In the process of integrating different datasets from a variety of sources, we verified that batch effects did not overwhelm the biological signal. Batch effects may derive not only from differences across datasets but also may be consequent of a different sequencing technique (PolyA+; TotalRNA; HCS) or originate from other unknown sources. We aimed at specifically removing technical batches rather than real biological variation and tried to preserve biological differences that may be consequent of a different PSA level, age, tumor grade/stage, or other. PCA, by identifying the transcriptional features endowed with the highest variance across samples, is a very useful tool to detect relevant batch effects. When the latter are overwhelming, they are likely to appear among the top PCs and cluster together samples sharing the same batch-effect-related features. A PCA analysis performed on the complete set of 1223 samples (Supplementary Fig. S1B) showed that the largest source of batch effects was associated with the HCS technique, while no relevant differences could be clearly associated with the dataset of origin. Only two of the CRPC datasets (phs000915 and phs000673) contained samples sequenced using HCS, and for several of these, matched technical replicates sequenced using PolyA+ technology were also available. This allowed us to assess and remove technology-associated bias in gene expression (ComBat algorithm, sva package v3.38.0, PolyA+ samples set as reference batch). We further reduced the possibility of confounding biological with technical variation by generating a training subset of our data, consisting of 883 PolyA+ samples (52 normal prostate, 620 primary tumors, 193 CRPCs, 19 NEPCs) and determined the top 2000 genes showing the highest amount of variation within the PolyA+ training set only. This way, for PCA representation, we avoid the selection of genes that are possibly affected by the sequencing technique, despite the correction we had already performed on the data. Hence, we used the same 2000 genes to generate a PCA plot computed on the extended set of samples. The PCA is routinely generated using the most variable genes detected across the entire dataset. DESeq2’s defaults are set to use the top 500 most variable genes only. This number is frequently applied when analyzing the transcription of protein-coding genes. Conversely, in our scenario we evaluated the expression of the comprehensive genomic annotations provided by Gencode, which also includes non-protein-coding genes, reaching a total amount of ~60,000 genes. Thus, we increased the number of genes used for PCA analysis proportionally to the above-mentioned number (4 × 500 = 2000).

The results depicted in the PCA plot shown in Fig. 1a clearly show that the positioning of tumors at the same stages of cancer progression overlap with each other irrespectively of the dataset of origin and the sequencing technology. This indicates that the different positioning of normal prostate, primary tumors, CRPCs, and NEPCs is due to a real biological signal and not consequent to an unwanted dataset-specific batch effect.

#### Integration and validation of additional bulk RNA-seq samples and pseudotime inference

We developed a method to include new prostate tumor samples in our current analysis by starting from raw counts, which allows the computation of pseudotime and PCs without modifying the original data and plots. Ideally, RNA-seq should be quantified using the sample genome (hg38) and references used for the current study (Gencode v29). Predictions can be performed sequentially, one sample at a time. For each new sample of interest, raw counts will be merged with the ones composing our full set. The obtained numeric matrix (the original matrix + 1 extra sample of interest) undergoes the same normalization and processing steps up to the computation of the PCA. Here, coordinates may slightly differ from the original ones, due to the adding of a new sample that might exert a small effect on the global re-normalization of all samples. To address this behavior, we apply a machine learning-based approach (glmnet package v4.1) that generates at runtime three elastic net models, one for each of the top 3 PCs, and train them to predict the error between the original coordinates and ones that are recomputed following the addition of the extra sample of interest. Hence, we apply these models to adjust the computed PC1, PC2, and PC3 coordinates of the extra sample, which can now be added to the PCA plot and pseudotime can be determined using slingshot.

#### Trajectory analysis

Trajectory and pseudotime inference are frequently used in scRNA-seq data analysis to model developmental trajectories through smooth curves following dimensionality reduction and clustering. Here, we applied one of these tools, slingshot (v1.6.0), to infer progression-associated trajectory and pseudotime from our integrated set of bulk RNA-seq samples. We selected slingshot because of its capability to also determine branches along the trajectory if any. PCA positioning (PC1–PC2) of the individual samples was used as input for slingshot, along with the information that the computed trajectory had to start from the normal tissue cluster. The analysis was performed using 1106 samples, discarding all technical replicates, in order not to overweight some samples and influence the computation of the trajectory. Metastatic lesions from the same individual but localized in different organs were admitted for this analysis. Subsequently, we could associate a pseudotime for each sample, ranging from 0 to 250 (Fig. 1b).

#### Correlation of genes and pathways to pseudotime

Having defined a unique pseudotime value for each sample, we computed the correlation between pseudotime and mRNA expression for each gene. For this purpose, we used Pearson’s correlation over Spearman’s because we aimed at identifying the strength of the linear relationship between gene expression and pseudotime. However, to be more robust to outliers, we opted for ten times repeated leave one-third out procedure. Precisely, we randomly selected ten subsets composed of 66% of the samples and computed correlation coefficients between pseudotime and expression of each gene in all subsets. Finally, we averaged these values and ranked them according to their correlation coefficient to pseudotime. Subsequently, using this ranking we applied Camera to perform GSEA procedure (use.ranks = TRUE) and determined which gene sets were mostly directly or inversely associated with pseudotime (Supplementary Fig. S1F).

#### Correlation of mRNA expression and protein abundances

Proteomics data were retrieved from the Proteomics Identifier Database (PRIDE: projects PXD009868, PXD003430, PXD003452, PXD003515, PXD004132, PXD003615, PXD003636, see “Data availability” section). The dataset includes 28 gland-confined prostate tumors and 8 adjacent non-malignant prostate tissues obtained from radical prostatectomy procedures, plus 22 bone metastatic prostate tumors obtained from patients operated to relieve spinal cord compression. To compute the correlation between mRNA expression and protein abundance, we first computed, for each gene, the average FC (log_2_) between CRPC and primary tumors based on mRNA expression. Then, the same was applied to the proteomics data to obtain for each protein a log FC representing differential abundance between CRPCs and primary tumors. For protein/mRNA correlation purposes, we discarded all genes that had not been evaluated in the proteomic data. Finally, we used Pearson’s method to evaluate the strength of correlation and the associated statistical significance.

#### Retrieval of genetic information and correlation with progression

Matched genetic information respective to mutations and copy-number status could be retrieved for 763 samples through cBioportal. Samples for which this information was available are indicated in Supplementary Data 1. To determine associations between mutations and tumor progression, for each gene we compared the pseudotime of mutant versus wild-type samples, by performing statistical testing using Wilcoxon’s rank-sum test (two-tailed). Mutations were ordered according to their FDR-adjusted *P* values and analyses were performed separately in primary and CRPC + NEPC tumors, to determine the relative contribution of mutations at various stages of disease progression. We only screened for genes being mutated in more than five individuals (Supplementary Fig. S1L). To determine associations between copy-number alterations and tumor progression, we associated for each gene a value of either −2 (homozygous deletion), −1 (heterozygous deletion), 0 (wild type), 1 (gain), 2 (amplification), and subsequently computed Pearson’s correlation between these values and pseudotime. We restricted this last analysis to genes being frequently deleted or amplified in prostate tumors, namely, *MYC*, *AR*, *RB1*, *PTEN*, and *TP53* (Fig. 1e). The above-described analyses were performed discarding technical replicates. Metastatic lesions from the same individual but localized in different organs were admitted for this analysis.

#### Quantification of immune infiltrates and correlation with progression

Quantification of immune infiltrates for all samples in our cohort was inferred from transcriptomic data using CibersortX^77^ by using the default signature matrix “LM22” to deconvolve 22 immune cell subsets from bulk RNA-seq (absolute quantification mode). The abundance of inferred immune populations was correlated to pseudotime using the same strategy applied to correlate gene expression and pseudotime. We opted for ten times repeated leave one-third out procedure. Precisely, we randomly selected ten0 subsets composed of 66% of the samples and computed correlation coefficients between pseudotime and each immune population in all subsets. Finally, we averaged these values and ranked them according to their correlation coefficient to pseudotime. Pearson’s correlation-associated *P* values were corrected for multiple testing using the FDR.

#### Macrophage polarization index

The macrophage polarization index, indicating polarization towards M1 or M2 phenotypes, was computed for all bulk RNA samples in our cohort using MacSpectrum^78–83^.

### scRNA-seq data processing

#### Quantification of gene expression

*Fastq* files were generated by demultiplexing raw data using cellranger (v3.1.0). To make single-cell gene-expression quantification more comparable to those of bulk RNA-seq, we generated a custom genome with cellranger, using the very same reference (GRCh38.p12) and annotations (Gencode v29) we had used for STAR when performing bulk RNA-seq analysis. To discriminate between human and murine cells that may infiltrate the tumors in the in vivo setting, we created a Mouse-Human reference, by creating a hybrid genome (GRCh38.p12 + GRCm38.p6) and hybrid gene-annotations (Gencode v29 and M25, for human and mouse genes, respectively). To avoid conflicts, mouse genomic coordinates were preceded by a prefix (i.e., mm_chr1, mm_chr2, etc.). Subsequently, cellranger was used to quantify gene expression in the form of an h5 filtered matrix where Ensembl gene IDs are used as identifiers.

#### Data filtering and clustering

Expression quantification files were imported in R statistical environment using Seurat (v3.1.5) package. We discarded individual cells from our data matrix by using two filtering procedures: first, we aimed at detecting transcriptional outliers, and second, we looked for putative doublets, which we also discarded. Briefly, we computed per-cell quality control metrics using scater (v1.16.1). The total amount of mitochondrial and ribosomal gene expression was quantified for both human and mouse cells. The number of genes being detected per cell, the total amount of reads per cell and the mitochondrial and ribosomal fraction of the transcriptome were used to determine the skewness-adjusted multivariate outlyingness for each cell (robustbase v0.93-6). Outliers were detected by median absolute deviation and removed at both tails. Counts were then normalized (Seurat::NormalizeData, method = LogNormalize, scale.factor = 1000) and the top 2000 most variable features were selected (Seurat::FindVariableFeatures, method = vst). Data were then scaled (Seurat::ScaleData) and PCA was performed up to the top 50 components (Seurat::RunPCA). Subsequently, we identified and eliminated putative doublets using DoubletFinder (v2.0.3). Having identified outliers and doublets, we removed them from the original count data and went through the preprocessing step again (i.e., normalization, scaling, and pca reduction). We proceeded to the determination of the *k*-nearest neighbors of each cell and the construction of a shared nearest-neighbor (SNN) graph (Seurat::FindNeighbors), then we identified clusters using the SNN modularity optimization-based clustering algorithm (Seurat::FindClusters, resolution = 0.5). Finally, we performed Umap dimensionality reduction on the first ten PCs, annotated the previously identified clusters, and generated plots accordingly.

#### Identification of cell cycle phase and cell type

We retrieved the list of cell cycle markers^84,85^ and subdivided it into markers of G2/M phase or S phase, according to Seurat’s annotations. We then used this information to infer the cell cycle phase in our samples (Seurat::CellCycleScoring). Murine cells could be clearly distinguished from human cancer cells, because of the intrinsic differences that could be easily spotted owing to the alignment and quantification performed using a hybrid human-mouse genome. Murine cell types were identified using SingleR (v1.2.4)^86^, using ImmGen repository^87^.

#### Dealing with drop-out events

Drop-out events are very frequent in the single-cell experiment performed using 10x Chromium technology. To address these issues, we applied Markov affinity-based graph imputation of cells (RMagic v2.0.3)^88^.

#### Differential expression analysis and gene-set enrichment

Differential expression was performed between different cell clusters and between clusters subjected to different treatment conditions (Seurat::Findmarkers) using a hurdle model tailored to scRNA-seq data (MAST method). Genes were subsequently ranked for log_2_ FC, and the Camera algorithm (pre-ranked) was used to determine gene-set enrichments for each comparison. Cell-specific gene-set enrichments were determined using single-sample GSEA, computed using gene-expression values of each cell following RMagic imputation.

#### Macrophage polarization index of macrophages

The macrophage polarization index, indicating polarization towards M1 or M2 phenotypes was computed for all cells being identified as macrophages from SingleR analysis (https://macspectrum.uconn.edu).

#### Macrophage reclustering

We could identify a sustained number of murine macrophages infiltrating all xenograft models, except for PNPCa cells. We isolated them and performed a cell-type-specific analysis by repeating all previously described processing steps (i.e., normalization, scaling, and pca reduction). Drop-out events were addressed using RMagic, and cell-specific enrichments were computed using a single-sample GSEA.

#### Integration of scRNA-seq with bulk RNA samples, PCA, and pseudotime inference

Single-cell experiments can be easily integrated with bulk RNA experiments by simply summing up together gene counts for all individual cells into one meta-element. This has proven to be comparable in terms of pseudotime inference and PCA positioning, as scRNA-seq and bulk RNA-seq experiments performed on the same cells are overimposable to each other. The same applies for the integration of single-cell-derived clusters, provided that the number of cells composing each cluster is not so critically low that the number of drop-out events results in a matrix composed of too many missing genes. If this is the case, or if just a single cell is to be integrated into the analysis, we suggest running RMagic to deal with the drop-out events, and then simply proceed as previously described.

### Additional resources

#### PCaProfiler

We provide a resource for the research community endowed with a web-based interface to facilitate the mining of the Prostate Cancer Transcriptome Atlas, called the PCaProfiler (https://www.prostatecanceratlas.org/). Using this resource, scientists can easily interrogate the atlas, recapitulate the findings shown in this study, and extend these by exploiting correlations between genes of interest and prostate cancer progression. PCaProfiler will allow integration and pseudotime inference of new cancer transcriptomes that the user can directly upload, compute, and visualize on the server. All results can be downloaded and re-uploaded to PCaProfiler when needed. Preloaded are PCA positioning and pseudotime inferences of the cell line, xenografts, and organoid models, as well as single-cell clusters and additional transcriptional datasets not included in the current study (i.e., PRJEB25542, ESCAPE Trial). PCaProfiler will be updated frequently with new data as new samples are being released or under specific requests.

#### Quantification and statistical analysis

Quantification methods and statistical analysis methods were described and referenced in the respective “Method details” subsection. If not otherwise specified, all statistical tests were corrected for multiple comparisons using the FDR correction method.

### Reporting summary

Further information on research design is available in the Nature Research Reporting Summary linked to this article.

## Supplementary information

Supplementary Information

Description of Additional Supplementary Files

Supplementary Data 1

Supplementary Data 2

Supplementary Data 3

Reporting Summary

## Source data

Source Data

## Data Availability

The bulk RNA-seq data generated in this study have been deposited in the EMBL-EBI database under accession code E-MTAB-9930. The single-cell RNA-seq data generated in this study for LuCaP PDX models and LNCaP cells have been deposited in the EMBL-EBI database under accession code E-MTAB-9903. The publicly available RNA-seq data used in this study are available in GEO (Gene Expression Omnibus), SRA (Short Read Archive), and EMBL-EBI databases under accession codes GSE120795^23^, GSE120741^19^, GSE118435^22^, GSE126078^21^, PRJNA477449^89^, PRJEB21092^90^, and E-MTAB-9656. The Proteomics data used in this study are available in the PRIDE database under accession codes PXD009868, PXD003430, PXD003452, PXD003515, PXD004132, PXD003615, and PXD003636. A minimum dataset to reproduce our findings containing vst-normalized expression data, along with its annotations, was made available (Zenodo repository, 10.5281/zenodo.5546618). All the software used for the analyses is described and referenced in the respective “Method details” subsections. All gene sets used for enrichment analyses were retrieved from the Molecular Signature Database (MSigDB). Source data are provided with this paper.

## References

[1] Vogelstein, B. et al. Cancer genome landscapes. *Science* **339**, 1546–1558 (2013). 23539594 10.1126/science.1235122 PMC3749880

[2] Garraway, L. A. & Lander, E. S. Lessons from the cancer genome. *Cell* **153**, 17–37 (2013). 23540688 10.1016/j.cell.2013.03.002

[3] Beltran, H. et al. Whole-exome sequencing of metastatic cancer and biomarkers of treatment response. *JAMA Oncol.* **1**, 466–474 (2015). 26181256 10.1001/jamaoncol.2015.1313 PMC4505739

[4] Yaeger, R. et al. Clinical sequencing defines the genomic landscape of metastatic colorectal cancer. *Cancer Cell* **33**, 125–136.e123 (2018). 29316426 10.1016/j.ccell.2017.12.004 PMC5765991

[5] Janjigian, Y. Y. et al. Genetic predictors of response to systemic therapy in esophagogastric cancer. *Cancer Discov.* **8**, 49–58 (2018). 29122777 10.1158/2159-8290.CD-17-0787 PMC5813492

[6] Zehir, A. et al. Mutational landscape of metastatic cancer revealed from prospective clinical sequencing of 10,000 patients. *Nat. Med.* **23**, 703–713 (2017). 28481359 10.1038/nm.4333 PMC5461196

[7] Robinson, D. R. et al. Integrative clinical genomics of metastatic cancer. *Nature* **548**, 297–303 (2017). 28783718 10.1038/nature23306 PMC5995337

[8] Morris, L. G. T. et al. The molecular landscape of recurrent and metastatic head and neck cancers: insights from a precision oncology sequencing platform. *JAMA Oncol.* **3**, 244–255 (2017). 27442865 10.1001/jamaoncol.2016.1790 PMC5253129

[9] Lefebvre, C. et al. Mutational profile of metastatic breast cancers: a retrospective analysis. *PLoS Med.* **13**, e1002201 (2016). 28027327 10.1371/journal.pmed.1002201 PMC5189935

[10] Armenia, J. et al. The long tail of oncogenic drivers in prostate cancer. *Nat. Genet.* **50**, 645–651 (2018). 29610475 10.1038/s41588-018-0078-z PMC6107367

[11] Robinson, D. et al. Integrative clinical genomics of advanced prostate cancer. *Cell* **161**, 1215–1228 (2015). 26000489 10.1016/j.cell.2015.05.001 PMC4484602

[12] Demircioglu, D. et al. A Pan-cancer Transcriptome Analysis reveals pervasive regulation through alternative promoters. *Cell* **178**, 1465–1477.e1417 (2019). 31491388 10.1016/j.cell.2019.08.018

[13] Ma, X. et al. Pan-cancer genome and transcriptome analyses of 1,699 paediatric leukaemias and solid tumours. *Nature* **555**, 371–376 (2018). 29489755 10.1038/nature25795 PMC5854542

[14] Dhanasekaran, S. M. et al. Delineation of prognostic biomarkers in prostate cancer. *Nature* **412**, 822–826 (2001). 11518967 10.1038/35090585

[15] Varambally, S. et al. The polycomb group protein EZH2 is involved in progression of prostate cancer. *Nature* **419**, 624–629 (2002). 12374981 10.1038/nature01075

[16] Kumar, A. et al. Substantial interindividual and limited intraindividual genomic diversity among tumors from men with metastatic prostate cancer. *Nat. Med.* **22**, 369–378 (2016). 26928463 10.1038/nm.4053 PMC5045679

[17] Beltran, H. et al. Divergent clonal evolution of castration-resistant neuroendocrine prostate cancer. *Nat. Med.* **22**, 298–305 (2016). 26855148 10.1038/nm.4045 PMC4777652

[18] Consortium, G. T. The Genotype-Tissue Expression (GTEx) project. *Nat. Genet.* **45**, 580–585 (2013). 23715323 10.1038/ng.2653 PMC4010069

[19] Stelloo, S. et al. Integrative epigenetic taxonomy of primary prostate cancer. *Nat. Commun.* **9**, 4900 (2018). 30464211 10.1038/s41467-018-07270-2 PMC6249266

[20] Lapuk, A. V. et al. From sequence to molecular pathology, and a mechanism driving the neuroendocrine phenotype in prostate cancer. *J. Pathol.* **227**, 286–297 (2012). 22553170 10.1002/path.4047 PMC3659819

[21] Labrecque, M. P. et al. Molecular profiling stratifies diverse phenotypes of treatment-refractory metastatic castration-resistant prostate cancer. *J. Clin. Invest.* **129**, 4492–4505 (2019). 31361600 10.1172/JCI128212 PMC6763249

[22] Sharp, A. et al. Androgen receptor splice variant-7 expression emerges with castration resistance in prostate cancer. *J. Clin. Invest.* **129**, 192–208 (2019). 30334814 10.1172/JCI122819 PMC6307949

[23] Suntsova, M. et al. Atlas of RNA sequencing profiles for normal human tissues. *Sci. Data* **6**, 36 (2019). 31015567 10.1038/s41597-019-0043-4 PMC6478850

[24] Abida, W. et al. Genomic correlates of clinical outcome in advanced prostate cancer. *Proc. Natl Acad. Sci. USA* **116**, 11428–11436 (2019). 31061129 10.1073/pnas.1902651116 PMC6561293

[25] Barbieri, C. E. et al. Exome sequencing identifies recurrent SPOP, FOXA1 and MED12 mutations in prostate cancer. *Nat. Genet.* **44**, 685–689 (2012). 22610119 10.1038/ng.2279 PMC3673022

[26] Cancer Genome Atlas Research, N. The molecular taxonomy of primary prostate cancer. *Cell* **163**, 1011–1025 (2015). 26544944 10.1016/j.cell.2015.10.025 PMC4695400

[27] Shoag, J. et al. SPOP mutation drives prostate neoplasia without stabilizing oncogenic transcription factor ERG. *J. Clin. Invest.* **128**, 381–386 (2018). 29202479 10.1172/JCI96551 PMC5749531

[28] Bernasocchi, T. et al. Dual functions of SPOP and ERG dictate androgen therapy responses in prostate cancer. *Nat. Commun.* **12**, 734 (2021). 33531470 10.1038/s41467-020-20820-x PMC7854732

[29] Gaytan de Ayala Alonso, A. et al. A genetic screen identifies novel polycomb group genes in Drosophila. *Genetics* **176**, 2099–2108 (2007). 17717194 10.1534/genetics.107.075739 PMC1950617

[30] Xu, K. et al. EZH2 oncogenic activity in castration-resistant prostate cancer cells is polycomb-independent. *Science* **338**, 1465–1469 (2012). 23239736 10.1126/science.1227604 PMC3625962

[31] Yu, J. et al. An integrated network of androgen receptor, polycomb, and TMPRSS2-ERG gene fusions in prostate cancer progression. *Cancer Cell* **17**, 443–454 (2010). 20478527 10.1016/j.ccr.2010.03.018 PMC2874722

[32] Wang, Q. et al. Androgen receptor regulates a distinct transcription program in androgen-independent prostate cancer. *Cell* **138**, 245–256 (2009). 19632176 10.1016/j.cell.2009.04.056 PMC2726827

[33] Pomerantz, M. M. et al. The androgen receptor cistrome is extensively reprogrammed in human prostate tumorigenesis. *Nat. Genet.* **47**, 1346–1351 (2015). 26457646 10.1038/ng.3419 PMC4707683

[34] Pomerantz, M. M. et al. Prostate cancer reactivates developmental epigenomic programs during metastatic progression. *Nat. Genet.* **52**, 790–799 (2020). 32690948 10.1038/s41588-020-0664-8 PMC10007911

[35] Iglesias-Gato, D. et al. The proteome of prostate cancer bone metastasis reveals heterogeneity with prognostic implications. *Clin. Cancer Res.* **24**, 5433–5444 (2018). 30042207 10.1158/1078-0432.CCR-18-1229

[36] Federer-Gsponer, J. R. et al. Patterns of stemness-associated markers in the development of castration-resistant prostate cancer. *Prostate* **80**, 1108–1117 (2020). 32628318 10.1002/pros.24039

[37] Di Mitri, D. et al. Re-education of tumor-associated macrophages by CXCR2 blockade drives senescence and tumor inhibition in advanced prostate cancer. *Cell Rep.* **28**, 2156–2168.e2155 (2019). 31433989 10.1016/j.celrep.2019.07.068 PMC6715643

[38] Kowal, J., Kornete, M. & Joyce, J. A. Re-education of macrophages as a therapeutic strategy in cancer. *Immunotherapy* **11**, 677–689 (2019). 31088236 10.2217/imt-2018-0156

[39] Barkal, A. A. et al. CD24 signalling through macrophage Siglec-10 is a target for cancer immunotherapy. *Nature* **572**, 392–396 (2019). 31367043 10.1038/s41586-019-1456-0 PMC6697206

[40] Karkampouna, S. et al. Patient-derived xenografts and organoids model therapy response in prostate cancer. *Nat. Commun.* **12**, 1117 (2021). 33602919 10.1038/s41467-021-21300-6 PMC7892572

[41] Nguyen, H. M. et al. LuCaP prostate cancer patient-derived xenografts reflect the molecular heterogeneity of advanced disease and serve as models for evaluating cancer therapeutics. *Prostate* **77**, 654–671 (2017). 28156002 10.1002/pros.23313 PMC5354949

[42] Pauli, C. et al. Personalized in vitro and in vivo cancer models to guide precision medicine. *Cancer Discov.* **7**, 462–477 (2017). 28331002 10.1158/2159-8290.CD-16-1154 PMC5413423

[43] Akamatsu, S. et al. The placental gene PEG10 promotes progression of neuroendocrine prostate cancer. *Cell Rep.* **12**, 922–936 (2015). 26235627 10.1016/j.celrep.2015.07.012

[44] Beshiri, M. L. et al. A PDX/Organoid Biobank of advanced prostate cancers captures genomic and phenotypic heterogeneity for disease modeling and therapeutic screening. *Clin. Cancer Res.* **24**, 4332–4345 (2018). 29748182 10.1158/1078-0432.CCR-18-0409 PMC6125202

[45] Gao, D. et al. Organoid cultures derived from patients with advanced prostate cancer. *Cell* **159**, 176–187 (2014). 25201530 10.1016/j.cell.2014.08.016 PMC4237931

[46] Stuart, T. et al. Comprehensive Integration of single-cell data. *Cell* **177**, 1888–1902.e1821 (2019). 31178118 10.1016/j.cell.2019.05.031 PMC6687398

[47] Berger, A. et al. N-Myc-mediated epigenetic reprogramming drives lineage plasticity in advanced prostate cancer. *J. Clin. Invest.* **129**, 3924–3940 (2019). 31260412 10.1172/JCI127961 PMC6715370

[48] Mu, P. et al. SOX2 promotes lineage plasticity and antiandrogen resistance in TP53- and RB1-deficient prostate cancer. *Science* **355**, 84–88 (2017). 28059768 10.1126/science.aah4307 PMC5247742

[49] Bonner, J. C. Regulation of PDGF and its receptors in fibrotic diseases. *Cytokine Growth Factor Rev.* **15**, 255–273 (2004). 15207816 10.1016/j.cytogfr.2004.03.006

[50] Hall, M. E. et al. Metastatic hormone-sensitive prostate cancer: current perspective on the evolving therapeutic landscape. *OncoTargets Ther.* **13**, 3571–3581 (2020). 10.2147/OTT.S228355 PMC7201221 32431511

[51] Kyriakopoulos, C. E. et al. Chemohormonal therapy in metastatic hormone-sensitive prostate cancer: long-term survival analysis of the Randomized Phase III E3805 CHAARTED Trial. *J. Clin. Oncol.* **36**, 1080–1087 (2018). 29384722 10.1200/JCO.2017.75.3657 PMC5891129

[52] de Bono, J. S. et al. Prednisone plus cabazitaxel or mitoxantrone for metastatic castration-resistant prostate cancer progressing after docetaxel treatment: a randomised open-label trial. *Lancet* **376**, 1147–1154 (2010). 20888992 10.1016/S0140-6736(10)61389-X

[53] Petrylak, D. P. et al. Docetaxel and estramustine compared with mitoxantrone and prednisone for advanced refractory prostate cancer. *N. Engl. J. Med.* **351**, 1513–1520 (2004). 15470214 10.1056/NEJMoa041318

[54] Gandaglia, G., Fossati, N., Suardi, N., Montorsi, F. & Briganti, A. STAMPEDE trial and patients with non-metastatic prostate cancer. *Lancet* **388**, 234–235 (2016). 27479564 10.1016/S0140-6736(16)31038-8

[55] Clarke, N. W. et al. Addition of docetaxel to hormonal therapy in low- and high-burden metastatic hormone sensitive prostate cancer: long-term survival results from the STAMPEDE trial. *Ann. Oncol.* **30**, 1992–2003 (2019). 31560068 10.1093/annonc/mdz396 PMC6938598

[56] Sweeney, C. J. et al. Chemohormonal therapy in metastatic hormone-sensitive prostate cancer. *N. Engl. J. Med.* **373**, 737–746 (2015). 26244877 10.1056/NEJMoa1503747 PMC4562797

[57] Tannock, I. F. et al. Docetaxel plus prednisone or mitoxantrone plus prednisone for advanced prostate cancer. *N. Engl. J. Med.* **351**, 1502–1512 (2004). 15470213 10.1056/NEJMoa040720

[58] Yu, J. et al. A polycomb repression signature in metastatic prostate cancer predicts cancer outcome. *Cancer Res.* **67**, 10657–10663 (2007). 18006806 10.1158/0008-5472.CAN-07-2498

[59] Xiao, L. et al. Epigenetic reprogramming with antisense oligonucleotides enhances the effectiveness of androgen receptor inhibition in castration-resistant prostate cancer. *Cancer Res.* **78**, 5731–5740 (2018). 30135193 10.1158/0008-5472.CAN-18-0941 PMC6191320

[60] Shankar, E. et al. Dual targeting of EZH2 and androgen receptor as a novel therapy for castration-resistant prostate cancer. *Toxicol. Appl Pharmacol.* **404**, 115200 (2020). 32805266 10.1016/j.taap.2020.115200

[61] Wu, C. et al. Inhibition of EZH2 by chemo- and radiotherapy agents and small molecule inhibitors induces cell death in castration-resistant prostate cancer. *Oncotarget* **7**, 3440–3452 (2016). 26657505 10.18632/oncotarget.6497 PMC4823118

[62] Kirk, J. S. et al. Top2a identifies and provides epigenetic rationale for novel combination therapeutic strategies for aggressive prostate cancer. *Oncotarget* **6**, 3136–3146 (2015). 25605014 10.18632/oncotarget.3077 PMC4413643

[63] Shan, J. et al. Targeting Wnt/EZH2/microRNA-708 signaling pathway inhibits neuroendocrine differentiation in prostate cancer. *Cell Death Discov.* **5**, 139 (2019). 31583122 10.1038/s41420-019-0218-y PMC6768854

[64] Luo, J. et al. LncRNA-p21 alters the antiandrogen enzalutamide-induced prostate cancer neuroendocrine differentiation via modulating the EZH2/STAT3 signaling. *Nat. Commun.* **10**, 2571 (2019). 31189930 10.1038/s41467-019-09784-9 PMC6561926

[65] Puca, L. et al. Patient derived organoids to model rare prostate cancer phenotypes. *Nat. Commun.* **9**, 2404 (2018). 29921838 10.1038/s41467-018-04495-z PMC6008438

[66] Ku, S. Y. et al. Rb1 and Trp53 cooperate to suppress prostate cancer lineage plasticity, metastasis, and antiandrogen resistance. *Science* **355**, 78–83 (2017). 28059767 10.1126/science.aah4199 PMC5367887

[67] Escamilla, J. et al. CSF1 receptor targeting in prostate cancer reverses macrophage-mediated resistance to androgen blockade therapy. *Cancer Res.* **75**, 950–962 (2015). 25736687 10.1158/0008-5472.CAN-14-0992 PMC4359956

[68] Karthaus, W. R. et al. Regenerative potential of prostate luminal cells revealed by single-cell analysis. *Science* **368**, 497–505 (2020). 32355025 10.1126/science.aay0267 PMC7313621

[69] Laudato, S., Aparicio, A. & Giancotti, F. G. Clonal evolution and epithelial plasticity in the emergence of AR-independent prostate carcinoma. *Trends Cancer* **5**, 440–455 (2019). 31311658 10.1016/j.trecan.2019.05.008 PMC6658113

[70] Linja, M. J. & Visakorpi, T. Alterations of androgen receptor in prostate cancer. *J. Steroid Biochem. Mol. Biol.* **92**, 255–264 (2004). 15663988 10.1016/j.jsbmb.2004.10.012

[71] Koivisto, P. et al. Androgen receptor gene amplification: a possible molecular mechanism for androgen deprivation therapy failure in prostate cancer. *Cancer Res.* **57**, 314–319 (1997). 9000575

[72] Antonarakis, E. S. et al. AR-V7 and resistance to enzalutamide and abiraterone in prostate cancer. *N. Engl. J. Med.* **371**, 1028–1038 (2014). 25184630 10.1056/NEJMoa1315815 PMC4201502

[73] Gaddipati, J. P. et al. Frequent detection of codon 877 mutation in the androgen receptor gene in advanced prostate cancers. *Cancer Res.* **54**, 2861–2864 (1994). 8187068

[74] Calcinotto, A. et al. IL-23 secreted by myeloid cells drives castration-resistant prostate cancer. *Nature* **559**, 363–369 (2018). 29950727 10.1038/s41586-018-0266-0 PMC6461206

[75] Wu, D. & Smyth, G. K. Camera: a competitive gene set test accounting for inter-gene correlation. *Nucleic Acids Res.* **40**, e133 (2012). 22638577 10.1093/nar/gks461 PMC3458527

[76] Bluemn, E. G. et al. Androgen receptor pathway-independent prostate cancer is sustained through FGF signaling. *Cancer Cell* **32**, 474–489.e476 (2017). 29017058 10.1016/j.ccell.2017.09.003 PMC5750052

[77] Steen, C. B., Liu, C. L., Alizadeh, A. A. & Newman, A. M. Profiling cell type abundance and expression in bulk tissues with CIBERSORTx. *Methods Mol. Biol.* **2117**, 135–157 (2020). 31960376 10.1007/978-1-0716-0301-7_7 PMC7695353

[78] Li, C. et al. Single cell transcriptomics based-MacSpectrum reveals novel macrophage activation signatures in diseases. *JCI Insight* **5**, 10.1172/jci.insight.126453 (2019). 10.1172/jci.insight.126453 PMC6542613 30990466

[79] Groner, A. C. et al. TRIM24 is an oncogenic transcriptional activator in prostate cancer. *Cancer Cell* **29**, 846–858 (2016). 27238081 10.1016/j.ccell.2016.04.012 PMC5124371

[80] Cyrta, J. et al. Role of specialized composition of SWI/SNF complexes in prostate cancer lineage plasticity. *Nat. Commun.* **11**, 5549 (2020). 33144576 10.1038/s41467-020-19328-1 PMC7642293

[81] Spahn, M. et al. Expression of microRNA-221 is progressively reduced in aggressive prostate cancer and metastasis and predicts clinical recurrence. *Int. J. Cancer* **127**, 394–403 (2010). 19585579 10.1002/ijc.24715

[82] Kuroda, H., Kutner, R. H., Bazan, N. G. & Reiser, J. Simplified lentivirus vector production in protein-free media using polyethylenimine-mediated transfection. *J. Virol. Methods* **157**, 113–121 (2009). 19114057 10.1016/j.jviromet.2008.11.021

[83] Drost, J. et al. Organoid culture systems for prostate epithelial and cancer tissue. *Nat. Protoc.* **11**, 347–358 (2016). 26797458 10.1038/nprot.2016.006 PMC4793718

[84] Kowalczyk, M. S. et al. Single-cell RNA-seq reveals changes in cell cycle and differentiation programs upon aging of hematopoietic stem cells. *Genome Res.* **25**, 1860–1872 (2015). 26430063 10.1101/gr.192237.115 PMC4665007

[85] Beltran, H. et al. Molecular characterization of neuroendocrine prostate cancer and identification of new drug targets. *Cancer Discov.* **1**, 487–495 (2011). 22389870 10.1158/2159-8290.CD-11-0130 PMC3290518

[86] Aran, D. et al. Reference-based analysis of lung single-cell sequencing reveals a transitional profibrotic macrophage. *Nat. Immunol.* **20**, 163–172 (2019). 30643263 10.1038/s41590-018-0276-y PMC6340744

[87] Shay, T. & Kang, J. Immunological Genome Project and systems immunology. *Trends Immunol.* **34**, 602–609 (2013). 23631936 10.1016/j.it.2013.03.004 PMC4615706

[88] van Dijk, D. et al. Recovering gene interactions from single-cell data using data diffusion. *Cell* **174**, 716–729.e727 (2018). 29961576 10.1016/j.cell.2018.05.061 PMC6771278

[89] Oberhuber, M. et al. STAT3-dependent analysis reveals PDK4 as independent predictor of recurrence in prostate cancer. *Mol. Syst. Biol.* **16**, e9247 (2020). 32323921 10.15252/msb.20199247 PMC7178451

[90] Ramnarine, V. R. et al. The long noncoding RNA landscape of neuroendocrine prostate cancer and its clinical implications. *Gigascience* **7**, 10.1093/gigascience/giy050 (2018). 10.1093/gigascience/giy050 PMC6007253 29757368

[91] Finak, G. et al. MAST: a flexible statistical framework for assessing transcriptional changes and characterizing heterogeneity in single-cell RNA sequencing data. *Genome Biol.* **16**, 278 (2015). 26653891 10.1186/s13059-015-0844-5 PMC4676162

