# Supplementary material for: Dynamic prostate cancer transcriptome analysis delineates the trajectory to disease progression
Source: Nat Commun. 2021 Dec 2;12:7033. doi: 10.1038/s41467-021-26840-5 (PMC8640014; doi:10.1038/s41467-021-26840-5)
Supplement: Supplementary file 1 — Supplementary Information [file 41467_2021_26840_MOESM1_ESM.pdf]

# SUPPLEMENTARY FIGURES

## SUPPLEMENTARY FIGURE 1

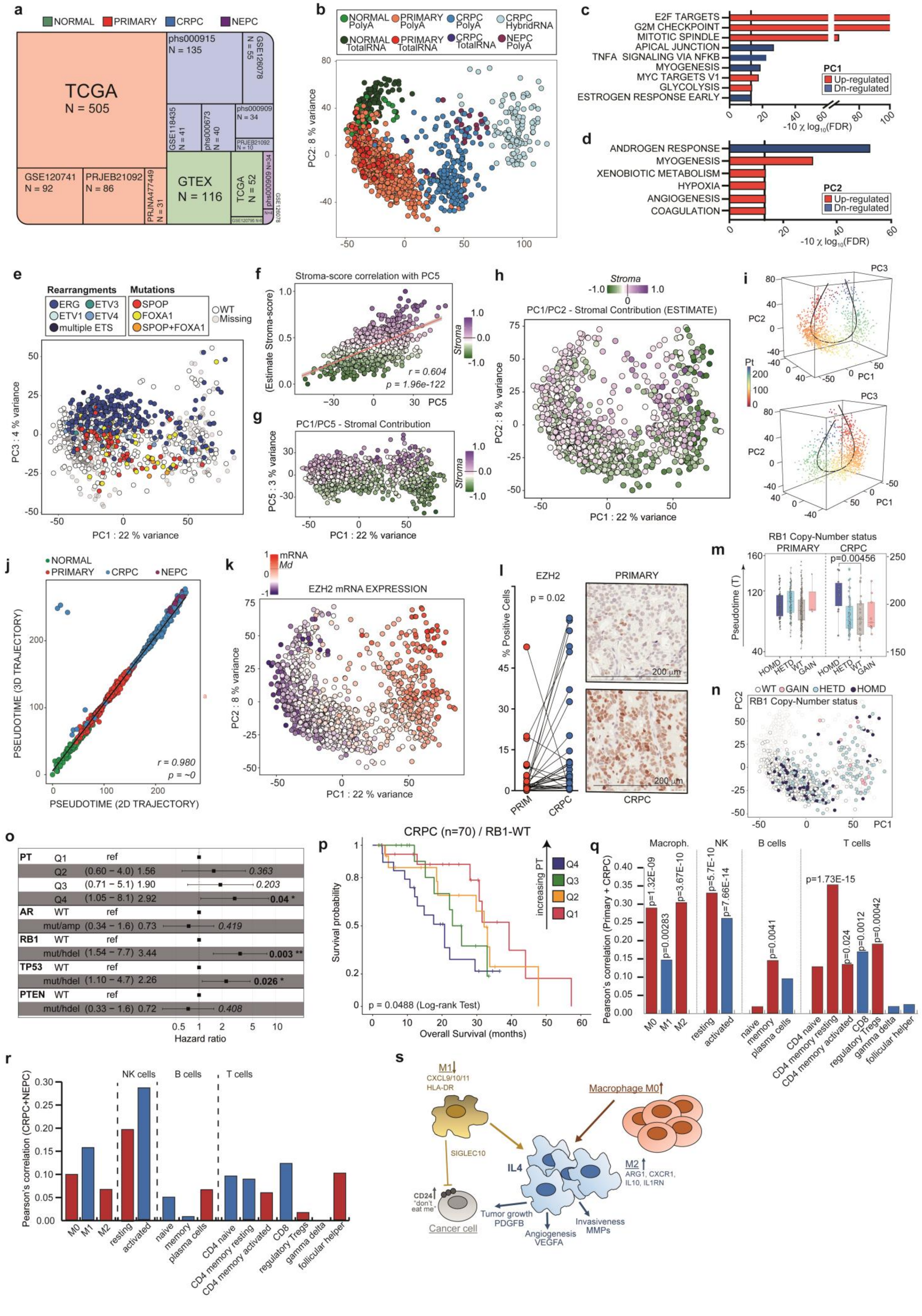

**Supplementary Figure 1.**

**(a)** Graphical representation of the RNA sequencing cohorts, their accession numbers, the total number of samples in each dataset, and tumor stages as indicated. Green: Normal Prostate; Red: Primary Tumors; Blue: Castration-resistant Tumors (CRPC); Violet: Neuro-endocrine Tumors (NEPC); See Source Data File. **(b)** Position of individual tumors in the PCA after re-processing of the raw data by selecting the top 2000 most variable genes. Hybrid capture-based RNA sequencing samples derived from CRPC are highlighted in light blue and show a marked but consistent shift along the PC1 and PC2 axes. No significant differences are observed in the first two principal components for TotalRNA when compared to PolyA+ samples. Samples/Sequencing-strategies are colored as indicated in the box on top of the figure panel. See Data Source File. **(c)** Gene-sets enrichments performed using Camera algorithm on genes ranked according to their relative contribution (coefficient) to the positioning of samples along the PC1 axis. The analysis performed on Hallmark gene sets reveals an increase of cell cycle-related gene sets along PC1. Blue: downregulated; Red: upregulated. See Data Source File. **(d)** Corresponding analysis performed on genes ranked according to their contribution to PC2 shows a decrease in androgen-responsive genes along this axis. Blue: downregulated; Red: upregulated. See Data Source File. **(e)** PCA plot representing the PC1/PC3 pane can be used to discern *SPOP*/*FOXA1* mutant prostate cancers from those harboring gene fusions involving ETS transcription factors. Genetic rearrangements and mutations are colored as indicated in the panel on top of the figure panel. **(f)** Stromal score correlation to the PC5 component. Pearson's correlation coefficient and associated p-value are reported. Stromal score was computed using ESTIMATE (Estimation of Stromal and Immune cells in Malignant Tumor tissues using Expression data). The score is represented in a three-color scale (green: lowest value; white: median value; violet: highest value). X-axis: the value of principal component 5 (PC5); Y-axis: The associated Estimate stroma-score, scaled between -1 and 1. See Source Data File. **(g)** Correlation between PC1 and PC5 values. The overall stroma-score across individual samples is also reported in a three-color scale (green: lowest value; white: median value; violet: highest value). X-axis: the value of principal component 1 (PC1); Y-axis: the value of principal component 5. The stroma-score is represented in a three-color scale (green: lowest value; white: median value; violet: highest value). See Source Data File. **(h)** Corresponding analysis does not show a major influence of the stromal component to the positioning of samples in the PC1-2 plot. X-axis: the value of principal component 1 (PC1); Y-axis: the value of principal component 2 (PC2); The stroma-score is represented in a three-color scale (green: lowest value; white: median value; violet: highest value), scaled between -1 and 1. See Source Data File. **(i)** Two distinct views of the 3-dimensional PCA plot with the corresponding 3D trajectory. X-axis: the value of principal component 1 (PC1); Y-axis: the value of principal component 2 (PC2); Z-axis: the value of principal component 3 (PC3); **(j)** Plot representing the correlation between the 2D- and 3D inferred pseudotime in the entire cohort (r = Pearson's correlation coefficient). **(k)** *EZH2* mRNA expression increases gradually along the main trajectory. Expression levels of each sample are reported within the PCA plot representing the PC1/PC2 pane. Gene expression levels are scaled between -1 and 1 and are represented in a three-color scale (blue: lowest value; white: median value; red: highest value). See Data Source File. **(l)** IHC analysis reveals upregulation of EZH2 in CRPC tumors compared to the matched primary tumors. Left: Quantification of EZH2 positive cells. Statistical significance was determined using two-sided Student's t-test (paired). Right: IHC images of a primary and its corresponding CRPC counterpart. See Source Data File. **(m)** Boxplots representing different pseudo-time distribution for RB1-specific copy number alterations in primary (homozygous n=79, heterozygous n=133, wild-type=262, gains=3) and CRPC samples (homozygous n=10, heterozygous n=53, wild-type=22, gains=10). P-values were computed using Wilcoxon's test (two-tailed) and adjusted using Bonferroni correction for 6 multiple comparisons (HOMD vs HETD, HOMD vs WT, HOMD vs GAIN both in CRPC and PRIMARY Tumor setting). Raw p-value = 0.00076; Bonferroni-adjusted p-value = 0.00456. Boxplots: Primary tumors, homozygous deletion: minimum = 59.2, lower-quartile = 79.21, median = 92.2, upper-quartile=109.4, maximum = 139.9); primary tumors, heterozygous deletion: minimum = 49.9, lower-quartile = 85.6, median = 101.9, upper-quartile= 116.1, maximum = 178.4); primary tumors, wild-type: minimum = 31.9, lower-quartile = 73.0, median = 87.58, upper-quartile= 103.18, maximum = 159.7; primary tumors, gains: minimum = 85.12, lower-quartile = 86.7, median = 88.2, upper-quartile= 114.1, maximum = 140.0). Castration-resistant (CRPC) tumors, homozygous deletion: minimum = 155.0, lower-quartile = 186.1, median = 205.2, upper-quartile= 225.1, maximum = 231.4); castration-resistant (CRPC) tumors, heterozygous deletion: minimum = 25.44, lower-quartile = 161.2, median = 174.9, upper-quartile= 189.0, maximum = 217.6); castration-resistant (CRPC) tumors, wild-type: minimum = 56.2, lower-quartile = 145.6, median = 169.5, upper-quartile= 182.7, maximum = 211.7; castration-resistant (CRPC) tumors, gains: minimum = 158.2, lower-quartile = 163.6, median = 167.2, upper-quartile= 199.2, maximum = 242.1). HOMD = darkblue; HETD = lightblue; WT = grey; GAIN = red. **(n)** Corresponding PCA plot highlighting *RB1* copy-number status acrosssamples. HOMD = darkblue; HETD = lightblue; WT = grey; GAIN = red. **(o)** Multivariate analysis using the Cox Proportional Hazard model shows the relative contribution of pseudotime, *AR* status, *RB1* status, *TP53* status and *PTEN* status to survival. Error bars represent 95% confidence intervals of the Hazard Ratio (HR). **(p)** Kaplan-Meier curve for overall

survival related to pseudotime using a 4-tiered scoring system (quartiles) in the subset of CRPCs patients with wild-tipe RB1. (p = 0.0488, Log-rank Test). Quartiles: Q1 (red); Q2 (orange); Q3 (green); Q4 (blue) **(q)** Histograms depicting the correlation between the inferred abundance of the indicated immune cell populations (as determined by Cibersortx) and pseudo-time in a subset composed of primary and CRPCs tumors. P-values associated with Pearson's correlation coefficients were adjusted for multiple testing using the false discovery rate (FDR) and reported on top of the bars. Red bars: positive correlation coefficient; blue bars: negative correlation coefficient. See Source Data File. **(r)** Histograms depicting the correlation between the inferred abundance of the indicated immune cell populations (as determined by Cibersortx) and pseudo-time in a subset composed of CRPCs and neuroendocrine tumors. See Source Data File. (**s)** Schematic representation of expression changes in genes linked to the tumor environment. Transcripts specific to M1-macrophages decrease along the trajectory while those of the M2 counterpart increase. M0 macrophages: orange; M1 macrophages: yellow; M2 macrophages: blue; Tumor cells: grey.

## SUPPLEMENTARY FIGURE 2

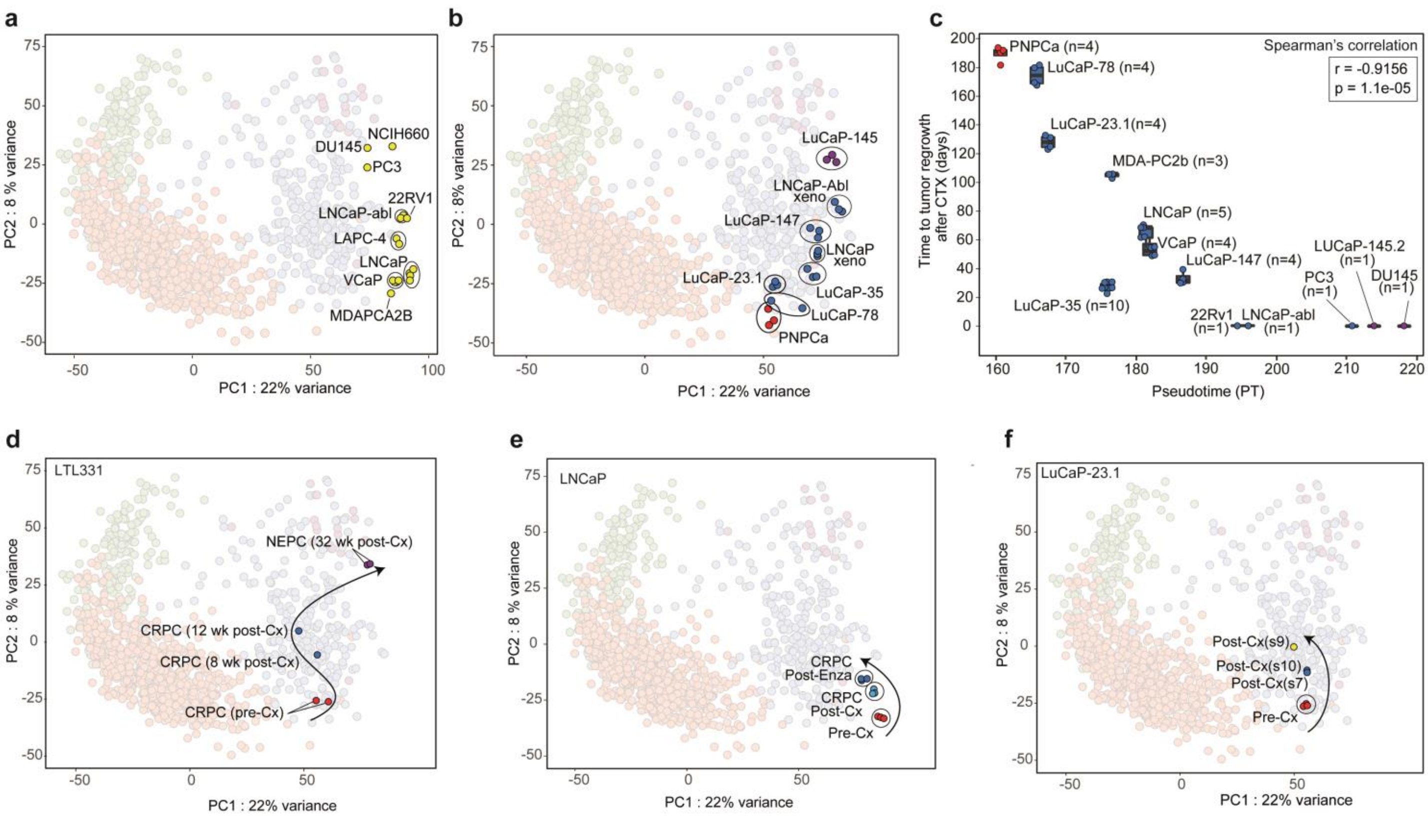


**Supplementary Figure 2.**

**(a)** Integration of the indicated ex-vivo cultured prostate cancer cell lines (in yellow) within the PCA plot. **(b)** Corresponding analysis for Xenografts. For the PNPCa model, the normal and primary tumor tissue's PCA position is reported and dramatically differs from the one found in immune-compromised mice. Red = Primary Tumor, Blue = CRPC, Violet = NEPC **(c)** Boxplots showing the correlation (Spearman, r = -0.9156/ p = 1.1e-05) between the pseudotime values inferred for each xenograft model and the corresponding latency of tumor regrowth after castration. X-axis: pseudotime value for the PDX and xenograft models; Y-axis: time to tumor regrowth after castration. Correlation with pseudotime was computed using the average latency for each xenograft: PNPCa=187.75; LuCaP-78=173; LuCaP23.1=127; LuCaP35=26.8; MDAPCA2B=104; LNCaP=64.20; VCaP=53.75; LuCaP147=33; 22Rv1, LNCaP-abl, PC3, LuCaP145.2 and DU145 = 0. Red = Primary Tumor, Blue = CRPC, Violet = NEPC. PNPCa (n=4 biologically independent experiments) : minimum = 180.0, lower-quartile = 186.7, median = 189.5, upper-quartile=190.5, maximum = 192.0); LuCaP-78 (n=4 biologically independent experiments) : minimum = 166.0, lower-quartile = 167.5, median = 173.0, upper-quartile=178.5, maximum = 180.0); LuCaP-23.1 (n=4 biologically independent experiments) : minimum = 122.0, lower-quartile = 123.5, median = 127.0, upper-quartile=130.5, maximum = 132.0); LuCaP-35 (n=10 biologically independent experiments) : minimum = 22, lower-quartile = 26, median = 26, upper-quartile=29, maximum = 30); MDA-PCa-2b (n=3 biologically independent experiments) : minimum = 102.0, lower-quartile = 103.5, median = 105, upper-quartile=105, maximum = 105); LNCaP (n=5 biologically independent experiments) : minimum = 54, lower-quartile = 61, median = 68, upper-quartile=68, maximum = 60); VCaP (n=4 biologically independent experiments) : minimum = 49, lower-quartile = 49, median = 52, upper-quartile=56.75, maximum = 62); LuCaP-147 (n=4 biologically independent experiments) : minimum =30.0, lower-quartile = 30.0, median = 31.5, upper-quartile=34.5, maximum = 39.0); 22Rv1, LNCaP-abl, PC3, LuCaP-145.2 and DU145 are DHT insensitive and don't stop growing after castration, thus 1 single experiment for each of these Xenograft model is reported, as their Time to Tumor regrowth equals 0. See Source Data. **(d-f)** After castration, the indicated PDX models and LNCaP xenograft progress along the main trajectory. Red = Primary Tumor, Blue = CRPC, Violet = NEPC.

## SUPPLEMENTARY FIGURE 3

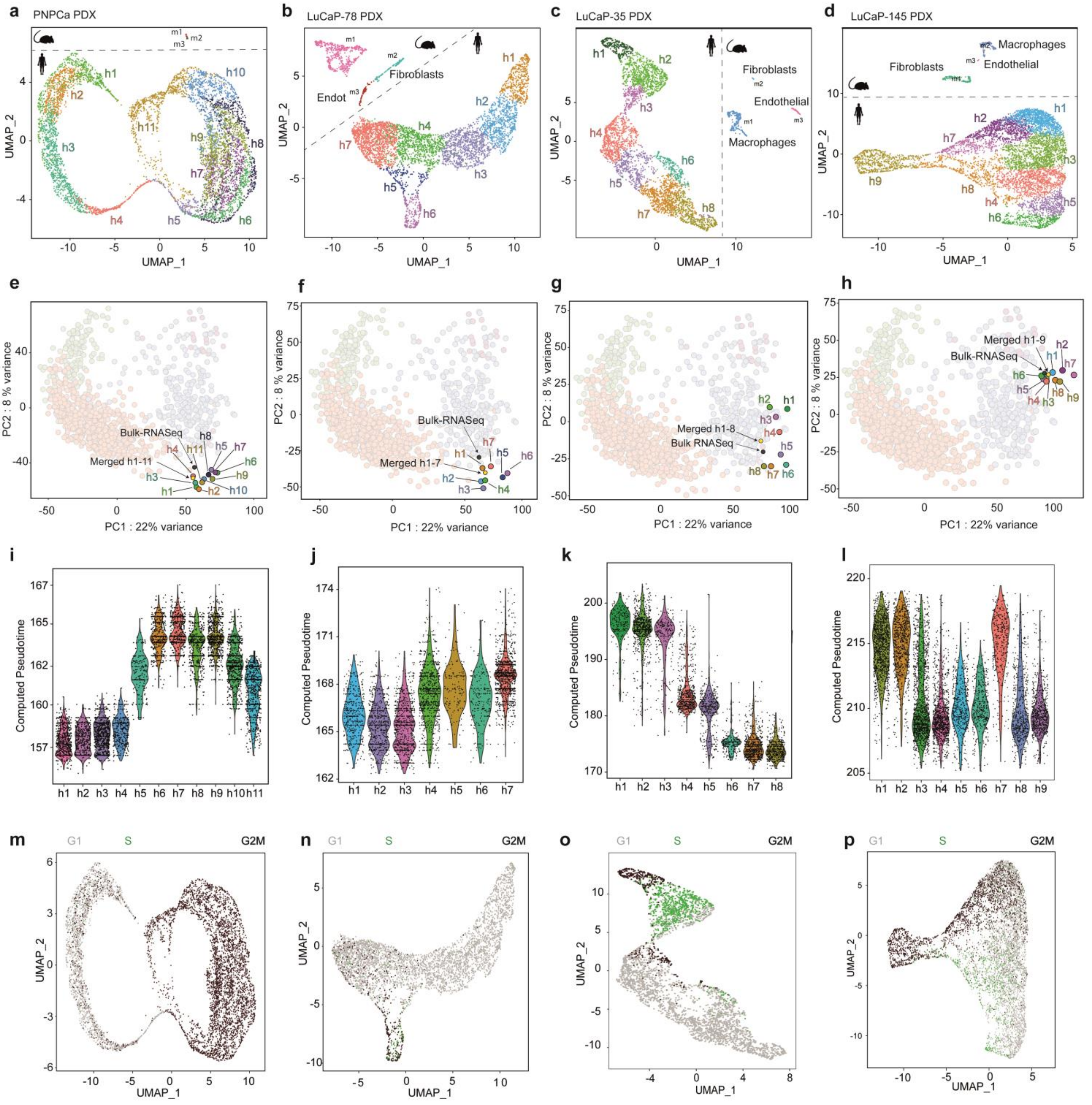


**Supplementary Figure 3.**

**(a-d)** Single-cell representation of the indicated PDX models in vivo using dimensionality reduction by Uniform Manifold Approximation and Projection (UMAP) and subsequent identification of tumor single-cell clusters using Seurat's workflow. Murine and human cell clusters are depicted in the indicated color. **(e-h)** The Integration of merged single-cell data of the indicated PDX model on the PCA plot shows a comparable position to the corresponding bulk RNA sequencing data. Individual single-cell clusters are also integrated into the PC1/PC2 pane. The highest dispersion of clusters along the main trajectory is seen for LuCaP-35. Human tumor cell clusters are depicted in the indicated color. **(i-l)** Violin plots indicating the pseudo-time of individual cells within the different cell clusters are depicted in the indicated color. The pseudo-time inference was performed following the imputation of missing genes (dropout events) by using RMagic. **(m-p)** Attribution of cells to either the G1, S, or G2M cellcycle phase for each PDX model. Cell Cycle Phase was determined using Seurat's workflow. S-Phase: green; G2M-Phase: brown; G1-Phase: light grey.

## SUPPLEMENTARY FIGURE 4

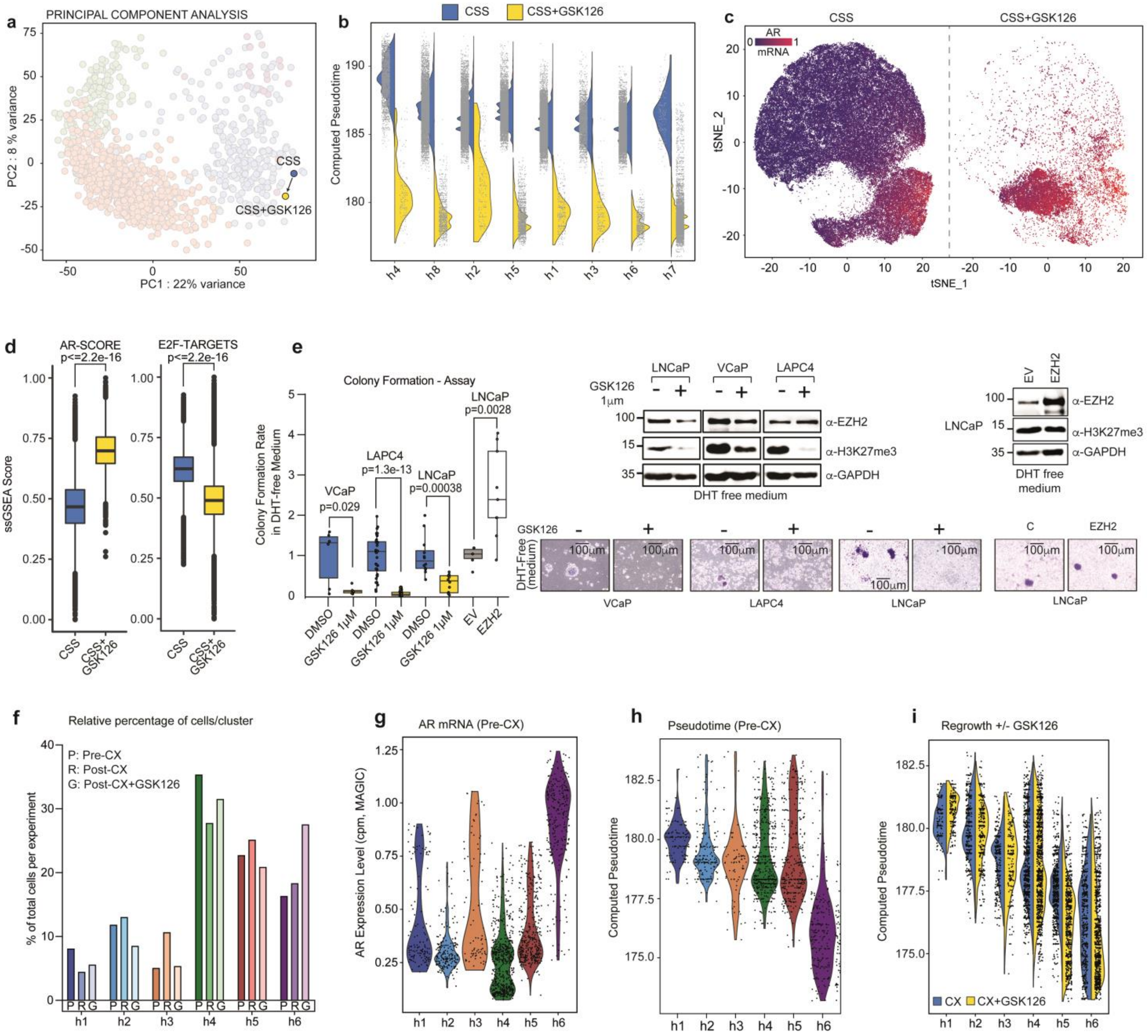

**Supplementary Figure 4.**

**(a)** Single-cell RNASeq data of LNCaP cells cultured in charcoal-stripped serum (CSS) was merged and integrated within the PCA plot (PC1/PC2). PCA positioning shows a decrease in pseudo-time upon EZH2 inhibition by GSK126. CSS: blue; CSS+GSK126: yellow. **(b)** Pseudotime of individual LNCaP cultured in charcoal-stripped serum (CSS) Is significantly reduced upon GSK126 treatment in each cell cluster (h1-h7). Pseudo-time was computed for each cell, following imputation of missing genes (drop-outs) using RMagic. Clusters are ordered according to decreasing pseudotime. CSS: blue; CSS+GSK126:yellow. **(c)** Dimensionality reduction (TSNE) of single-cell RNA-Seq performed on LNCaP cells cultured in vitro with charcoal-strippedserum (CSS) in the presence (right) or absence (left) of the EZH2 inhibitor GSK126. Upon GSK126 treatment, as most cell clusters disappear, there is an increase in *AR* mRNA expression in the transcriptionally rewired LNCaP cells that give rise to a novel cluster characterized by higher *AR* expression levels (depicted using the color scale indicated on the upper left of the figure panel). **(d)** Corresponding quantification (ssGSEA) of *AR*-SCORE and *E2F* target genes (Hallmark gene set) computed for each cell before (left) and following (right) EZH2 inhibition by GSK126. Missing gene-expression values (dropout events) for each cell were imputed using RMagic. P-values were computed using Wilcoxon's test (two-tailed) and adjusted for multiple testing using false discovery rate (FDR). *AR*-SCORE (left), CTRL (n=88600 cells): minimum = 0.00, lower-quartile = 0.40, median = 0.47, upper-quartile=0.54, maximum = 0.92); GSK126 (n=13713 cells): minimum = 0.26, lower-quartile = 0.65, median = 0.70, upper-quartile=0.76, maximum = 1.00); *E2F*-TARGETS (right), CTRL (n=88600 cells): minimum = 0.22, lower-quartile = 0. 57, median = 0.62, upper-quartile=0.67, maximum = 0.93); GSK126 (n=13713 cells): minimum = 0.00, lower-quartile = 0.43, median = 0.49, upper-quartile=0.55, maximum = 1.00); CSS: blue; CSS+GSK126: yellow. **(e)** GSK126 inhibits colony formation of LNCaP cells when cultured in CSS, while *EZH2* over-expression increases the number of

colonies under the same condition. Right, corresponding blots, molecular weights are annotated on the left. The membranes (from 12% gel) were cut based on protein molecular markers in different strips, and each of them was incubated with a specific antibody: strip-1(206-70 kD) with anti-EZH2; strip-2 (70-40KD) Not Reported; strip-3 (40-25KD) with anti-GAPH, and strip-4 (25-10k) with anti-H3K27me3. Left, P-values were determined using unpaired two-sided t-test and subsequently adjusted for multiple testing using FDR. VCaP-DMSO (n=6 biologically independent experiments) : minimum = 0.06, lower-quartile = 0.45, median = 1.33, upper-quartile=1.47, maximum = 1.60); VCaP-GSK (n=6 biologically independent experiments) : minimum = 0.059, lower-quartile = 0.08, median = 0.12, upper-quartile=0.14, maximum = 0.32); LAPC4-DMSO (n=36 biologically independent experiments) : minimum = 0.12, lower-quartile = 0.63, median = 1.11, upper-quartile=1.35, maximum = 1.98); LAPC4-GSK (n=36 biologically independent experiments) : minimum = 0.00, lower-quartile = 0.02, median = 0.05, upper-quartile=0.10, maximum = 0.22); LNCaP-DMSO (n=12 biologically independent experiments) : minimum = 0.41, lower-quartile = 0.69, median = 0.88, upper-quartile=1.13, maximum = 2.01); LNCaP-GSK (n=12 biologically independent experiments) : minimum = 0.03, lower-quartile = 0.09, median = 0.38, upper-quartile=0.51, maximum = 0.60); LNCaP-EV (n=6 biologically independent experiments) : minimum = 0.60, lower-quartile = 0.94, median = 1.05, upper-quartile=1.16, maximum = 1.20); LNCaP-EZH2 (n=9 biologically independent experiments) : minimum = 0.90, lower-quartile = 1.95, median = 2.40, upper-quartile=3.60, maximum = 4.05). DMSO: blue; GSK126: yellow; Empty Vector (EV): grey; EZH2-overexpressiong: white. See Source Data File. **(f)** Following EZH2 inhibition by GSK126, there is a common trend towards decreasing of cells in most clusters, except for cluster h6, which shows an opposite behavior. Relative percentage of cells per cluster (h1-h6) are shown for P: Pre-CX; R: Post-CX (Regrowth) and G: Post-CX + GSK126. Clusters (h1-h6) are depicted using different colors as indicated in the figure panel. P: high color saturation; R: intermediate color saturation; G: low color saturation. **(g)** Violin plots depicting AR expression levels show that the h6 cell cluster is characterized by higher levels of the latter. Missing gene-expression values (dropout events) for each cell were imputed using RMagic. Clusters (h1-h6) are depicted using different colors as indicated in the figure panel. **(h)** The pseudo-time inference was performed for each cell, and cluster h6 resulted to be associated with a less progressed phenotype. Clusters (h1-h6) are depicted using different colors as indicated in the figure panel. **(i)** Violin plots comparing pseudo-time before (blue) or following (yellow) EZH2 inhibition by GSK126. Cluster h6 displays the highest AR expression levels, the least progression on the main trajectory, and a reduction in pseudo-time after GSK126 treatment.

## SUPPLEMENTARY FIGURE 5

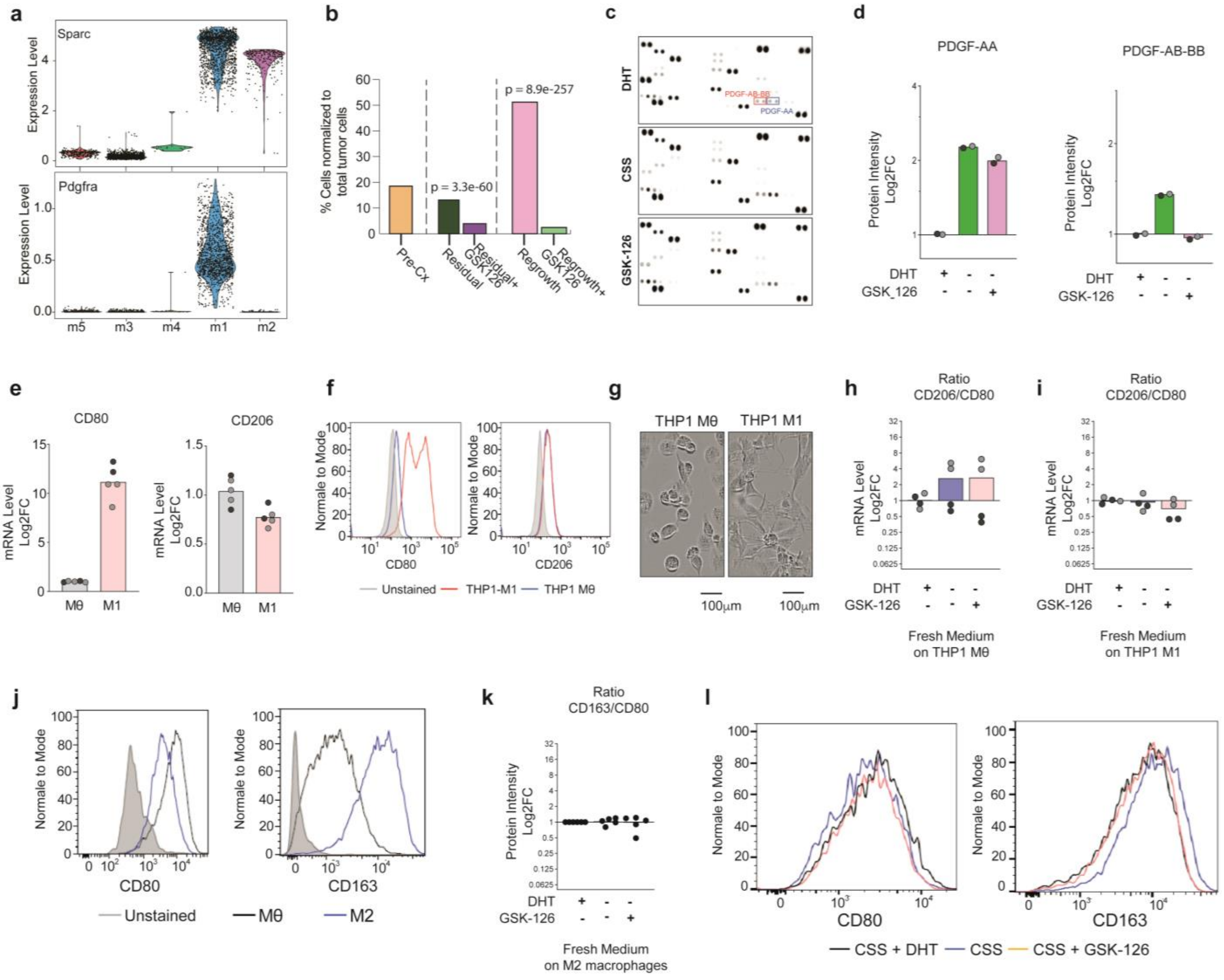


## Supplementary Figure 5

**(a)** Violin plots representing mRNA expression levels of two murine fibroblasts markers (Sparc and Pdgfra). Missing gene-expression values (dropout events) for each cell were imputed using RMagic. Murine clusters (m1-m5) are depicted using different colors. **(b)** Histogram representing the relative proportion of m1- cluster before castration (Pre-Cx), 80 days after castration (Residual; and with concomitant treatment with GSK126 inhibitor) and 120 days after castration (Regrowth; and with concomitant treatment with GSK126 inhibitor). P-values were determined using Chi-squared test and adjusted for multiple comparisons using Bonferroni correction. Pre-CX: yellow; Residual: dark green; Residual+GSK126: violet; Regrowth: pink; Regrowth+GSK126: light green. See Source Data File. **(c)** Cytokine Array Analysis of LNCaP Conditioned Media (CM). The protein expression profiles were evaluated in the supernatants of LNCaP maintained for 4 weeks in charcoal-stripped serum (CSS, middle panel), or CSS supplemented with 1 nM DHT (upper panel) or with1 µM GSK-126 (lower panel). Spots of PDGF-AA (blue box) and PDGF-AB-BB (red box) proteins are indicated with squares. Two technical replicates for each spot are included in the Array. **(d)** Bar graph showing the densitometric analysis of spots corresponding to PDGF-AA and PDGF-AB-BB proteins, present in CM of LNCaP maintained in DHT, CSS, and GSK-126. To calculate the Log2 Fold Changes (FC), the signaling (density pixel) of each spot was normalized to the array-specific positive control and then compared with the average signaling of the corresponding DHT condition. Bargraphs depict n=2 technical replicates from n=1 biologically independent cytokine array experiment as shown in C. Black and grey dots discriminate between technical replicates. CSS: green; CSS+GSK126: pink. See Source Data File. **(e-g)** Polarization of THP-1 cells into Mθ and M1 macrophages. The THP1-Mθ and the THP1-M1 macrophages were generated from THP1 cells after 48 h of stimulation with PMAS alone or in combination with LPS and INFY. **(e)** Bar graph showing the gene expression changing of the *CD80* (left panel, M1) and the *CD206* (right panel, M2) surface markers in THP-Mθ and THP-M1 macrophages. Gene expression levels were measured by RT-qPCR. The Log2 Fold Change was calculated using Actin as reference genes and compared to the Mθ macrophages (n=2 independent experiments per condition). Technical replicates are indicated in different colors. Experiment

1: black dots; Experiment 2: grey dots. Mθ: grey; M1: pink. See Source Data File. **(f)** Flow cytometry histograms showing surface marker expression of *CD80* and *CD206* in THP1-Mθ (red line), THP1-M1 (blue line), and in unstained controls (green line). Histograms depict the results obtain in one representative experiment. **(g)** Representative images of THP-1 cells polarized into Mθ (left) and M1 (right) macrophages. **(h-i)** Bar graph showing the $Log_2$ Fold Change Ratio of M2 versus M1 surface markers, in THP1-Mθ **(h)** or THP1-M1 **(i)** macrophages maintained for 72h in DHT, CSS, and GSK-126 fresh medium. The abundance of the CD80 (M1) and CD206 (M2) surface markers was determined by flow cytometry. The mean fluorescence intensity of the positive cells was used to determine the fold change ratio between the M2 and the M1 surface marker. N=2 independent biological experiments, with two technical replicates each. Black and grey dots discriminate between biological experiments. CSS: violet; CSS+GS126: pink. See Source Data File. **(j)** Differentiation of human CD14+ monocytes into M2-like macrophages. M2-like macrophages were generated from M-CSF and GM-CSF-treated human CD14+ cells stimulated with IL-13 and IL-4. The CD14+ cells treated only with M-CSF and GM-CSF were used as a comparison (unpolarized Mθ macrophages). Flow cytometry histograms showing the surface marker expression of CD80 (M1) and CD163(M2) in unpolarized Mθ macrophage (red line), M2-like macrophage (blue line), and the unstained controls (green line). Histograms depict the results obtain in one representative donor. **(k)** Bar graph showing the Log2 Fold Change Ratio of M2 (CD163) versus M1 (CD80) surface markers of M2-like macrophages polarized for 7 days in DHT, CSS, and GSK-126 fresh medium. Histograms depict the results obtain in five different donors. See Data Source File. **(l)** Flow cytometry histograms showing the surface marker abundance of CD80 (right graph) and CD163 (left marker) of M2-like macrophages polarized for 7 days with the conditioned medium of LNCaP maintained in CSS (blue line), 1 nM DHT (black line) or 1 µM GSK-126 (red line). Histograms depict the results obtain in one representative donor.

## SUPPLEMENTARY FIGURE 6

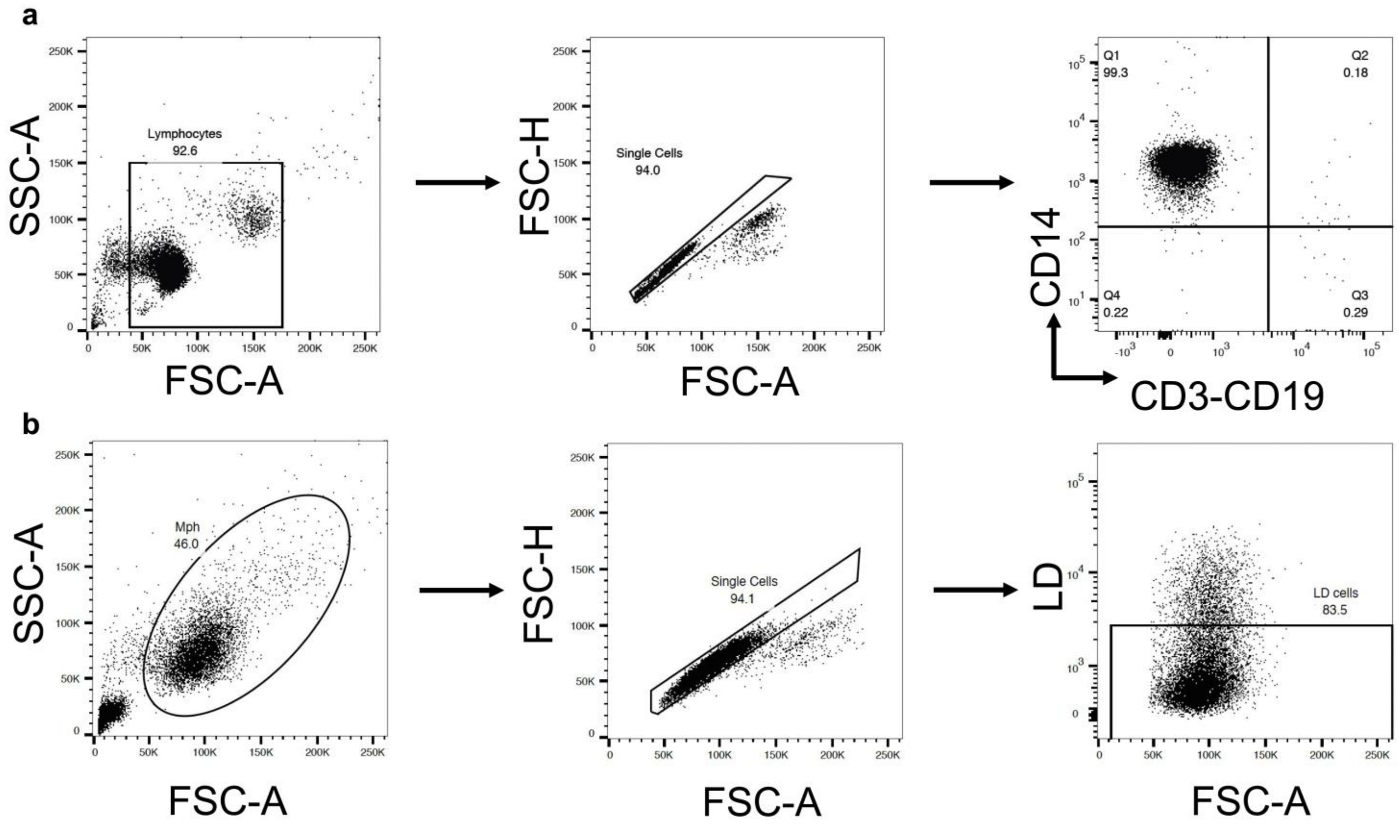


### Supplementary Figure 6

Purity checks of CD14+ enriched population. Representative flow cytometry analysis shows **(a)** the purity level of freshly isolated human CD14+cells upon isolation and **(b)** the gating strategy of monocytes-derived macrophages (day 7) treated in vitro with different cytokines to induce M0 or M1 or M2-like phenotype.
