# Supplementary material for: Dynamic prostate cancer transcriptome analysis delineates the trajectory to disease progression
Source: Nat Commun. 2021 Dec 2;12:7033. doi: 10.1038/s41467-021-26840-5 (PMC8640014; doi:10.1038/s41467-021-26840-5)
Supplement: Supplementary file 2 — Description of Additional Supplementary Files [file 41467_2021_26840_MOESM2_ESM.pdf]

Description of Additional Supplementary Files

File Name: Supplementary Data 1
Description: Clinical annotations

File Name: Supplementary Data 2
Description: Correlations between stroma-score and top 5 princial components

File Name: Supplementary Data 3
Description: List of Primers
