# Supplementary material for: Dynamic prostate cancer transcriptome analysis delineates the trajectory to disease progression
Source: Nat Commun. 2021 Dec 2;12:7033. doi: 10.1038/s41467-021-26840-5 (PMC8640014; doi:10.1038/s41467-021-26840-5)
Supplement: Supplementary file 6 — Reporting Summary [file 41467_2021_26840_MOESM6_ESM.pdf]

nature portfolio

Corresponding author(s): MARCO BOLIS, JEAN-PHILIPPE THEURILLAT

Last updated by author(s): Oct 8, 2021

Reporting Summary

Nature Portfolio wishes to improve the reproducibility of the work that we publish. This form provides structure for consistency and transparency in reporting. For further information on Nature Portfolio policies, see our Editorial Policies and the Editorial Policy Checklist.

Statistics

For all statistical analyses, confirm that the following items are present in the figure legend, table legend, main text, or Methods section.

n/a	Confirmed	
☐	☒	The exact sample size (*n*) for each experimental group/condition, given as a discrete number and unit of measurement
☐	☒	A statement on whether measurements were taken from distinct samples or whether the same sample was measured repeatedly
☐	☒	The statistical test(s) used AND whether they are one- or two-sided<br>*Only common tests should be described solely by name; describe more complex techniques in the Methods section.*
☐	☒	A description of all covariates tested
☐	☒	A description of any assumptions or corrections, such as tests of normality and adjustment for multiple comparisons
☐	☒	A full description of the statistical parameters including central tendency (e.g. means) or other basic estimates (e.g. regression coefficient) AND variation (e.g. standard deviation) or associated estimates of uncertainty (e.g. confidence intervals)
☐	☒	For null hypothesis testing, the test statistic (e.g. *F*, *t*, *r*) with confidence intervals, effect sizes, degrees of freedom and *P* value noted<br>*Give P values as exact values whenever suitable.*
☒	☐	For Bayesian analysis, information on the choice of priors and Markov chain Monte Carlo settings
☒	☐	For hierarchical and complex designs, identification of the appropriate level for tests and full reporting of outcomes
☐	☒	Estimates of effect sizes (e.g. Cohen's *d*, Pearson's *r*), indicating how they were calculated

Our web collection on statistics for biologists contains articles on many of the points above.

Software and code

Policy information about availability of computer code

Data collection	Depending on the specific dataset considered, fastq files were downloaded either by using gdc-client v1.6 (TCGA) or sra-tools v2.9.1 (SRA, dbGaP).
Data analysis	RNA-Sequencing: The overall quality of sequencing reads was evaluated using FastQC (v.0.11.9) (Andrews S., 2010). Sequence alignments to the reference human genome (GRCh38) were performed using STAR (v.2.6.1c) in two-pass mode, to significantly increase sensitivity to novel splice junctions compared to the regular single-mapping. In particular, gene expression was quantified at the gene level in the 2nd pass by using the comprehensive annotations made available by Gencode (v29 GTF-File). Strand-specific information was not maintained to avoid technical differences between stranded and unstranded libraries. Samples were adjusted far library size and normalized with the variance stabilizing transformation (vst) in the R statistical environment (v4.0.5) using the DESeq2 (v1.28.1) pipeline. When performing differential expression analysis between groups we applied the embedded IndependentFiltering procedure to exclude genes that were not expressed at appreciable levels in most of the samples considered. If not otherwise specified, all gene set enrichment analyses were performed using the limma (v.3.46.0) package (Camera, parametric test). Gene-Sets collections were retrieved either from the Molecular Signature Database (MSigDB), or from previous publications (AR/NE-Score). Batch effect correction was performed using the Combat algorithm (sva package v3.38.0). Training of machine learning models to predict PCA positioning and Pseutotime of new samples was performed using glmnet package (v4.1). Trajectory inference was performed using Slingshot (v.1.6.0).<br>Single-cell Sequencing: Raw files were demultiplexing using cellranger (v3.1.0). Quality control were performed using Scater (v1.16.1). Expression quantification files were imported in R (v4.0.5) statistical environment using Seurat (v3.1.5) package. Outlier detection was performed usinc Robustbase package (v0.93). Dublets were determined using DoubletFinder (v2.0.3). Murine cell types were identified using SingleR package (v1.2.4). Gene expression was imputed using RMagic package (v2.0.3).<br>Colony formation assays: Quantification of colonies was performed by ImageJ (v1.52p) software.

For manuscripts utilizing custom algorithms or software that are central to the research but not yet described in published literature, software must be made available to editors and reviewers. We strongly encourage code deposition in a community repository (e.g. GitHub). See the Nature Portfolio guidelines for submitting code & software for further information.

Data

Policy information about availability of data

All manuscripts must include a data availability statement. This statement should provide the following information, where applicable:

- Accession codes, unique identifiers, or web links for publicly available datasets
- A description of any restrictions on data availability
- For clinical datasets or third party data, please ensure that the statement adheres to our policy

The bulk RNA-Seq data generated in this study have been deposited in the EMBL-EBI database under accession code E-MTAB-9930 [https://www.ebi.ac.uk/arrayexpress/experiments/E-MTAB-9930]. The single-cell RNA-Seq data generated in this study for LuCaP PDX models and LNCaP cells have been deposited in the EMBL-EBI database under accession code E-MTAB-9903 [https://www.ebi.ac.uk/arrayexpress/experiments/E-MTAB-9903]. The publicly available RNA-Seq data used in this study are available in GEO (Gene Expression Omnibus), SRA (Short Rea Archive) and EMBL-EBI databases under accession codes GSE12079523 [https://www.ncbi.nlm.nih.gov/geo/query/acc.cgi?acc=GSE120795], GSE12074119 [https://www.ncbi.nlm.nih.gov/geo/query/acc.cgi?acc=GSE120741], GSE11843522 [https://www.ncbi.nlm.nih.gov/geo/query/acc.cgi?acc=GSE118435], GSE12607821 [https://www.ncbi.nlm.nih.gov/geo/query/acc.cgi?acc=GSE126078], PRJNA47744990 [https://www.ncbi.nlm.nih.gov/sra/?term=PRJNA477449], PRJEB2109291 [https://www.ncbi.nlm.nih.gov/sra/?term= PRJEB21092], E-MTAB-9656 [https://www.ebi.ac.uk/arrayexpress/experiments/E-MTAB-9656].
The Proteomics data used in this study are available in the PRIDE database under accession codes PXD009868 [https://www.ebi.ac.uk/pride/archive/projects/PXD009868], PXD003430 [https://www.ebi.ac.uk/pride/archive/projects/PXD003430], PXD003452 [https://www.ebi.ac.uk/pride/archive/projects/PXD003452], PXD003515 [https://www.ebi.ac.uk/pride/archive/projects/PXD003515], PXD004132 [https://www.ebi.ac.uk/pride/archive/projects/PXD004132], PXD003615 [https://www.ebi.ac.uk/pride/archive/projects/PXD003615], PXD003636 [https://www.ebi.ac.uk/pride/archive/projects/PXD003636].
A minimum dataset to reproduce our findings containing vst-normalized expression data, along with its annotations, was made available (Zenodo repository, DOI: 10.5281/zenodo.5546618). All the software used for the analyses is described and referenced in the respective Method Details subsections. All gene-sets used for enrichment analyses were retrieved from the Molecular Signature Database (MSigDB).

Field-specific reporting

Please select the one below that is the best fit for your research. If you are not sure, read the appropriate sections before making your selection.

☒ Life sciences ☐ Behavioural & social sciences ☐ Ecological, evolutionary & environmental sciences

For a reference copy of the document with all sections, see nature.com/documents/nr-reporting-summary-flat.pdf

Life sciences study design

All studies must disclose on these points even when the disclosure is negative.

Sample size	The samples size results from the available tissue of matched primary and CRPC samples at archives of the institute of pathologies in Basel.
Data exclusions	Data has not been excluded. In some cases data point are missing because of damage in tissue spots. This is a common issue with Tissue Microarrays (TMA).
Replication	All attempts of replications were successful. For each experiment, the number of biological independent experiments is reported in the figure legends.
Randomization	All samples/animals were randomly allocated to experimental groups and processed.
Blinding	Experiments were not blinded. However, we followed standard laboratory procedures of randomization. Each experiment was designed with proper controls, and samples for comparison were collected and analyzed under the same conditions.

Reporting for specific materials, systems and methods

We require information from authors about some types of materials, experimental systems and methods used in many studies. Here, indicate whether each material, system or method listed is relevant to your study. If you are not sure if a list item applies to your research, read the appropriate section before selecting a response.

Materials & experimental systems

n/a	Involved in the study
☐	☒ Antibodies
☐	☒ Eukaryotic cell lines
☒	☐ Palaeontology and archaeology
☐	☒ Animals and other organisms
☐	☒ Human research participants
☒	☐ Clinical data
☒	☐ Dual use research of concern

Methods

n/a	Involved in the study
☒	☐ ChIP-seq
☐	☒ Flow cytometry
☒	☐ MRI-based neuroimaging

Antibodies

Antibodies used

This information is also provided in tabular format in the Source Data File.

- anti-GAPDH Mouse/IgG, supplier: Santa Cruz, catalog number: sc-47724, dilution:(1:1000)
- anti-AR Mouse/IgG, supplier: Santa Cruz, catalog number: sc-7305, dilution:(1:500)
- anti-DNMT3A Mouse/IgG, supplier: Santa Cruz, catalog number: sc-365769, dilution:(1:500)
- anti-DNMT1 Rabbit/IgG, supplier: Cell Signaling Technologies), catalog number: 5032S, dilution:(1:1000)
- anti-EED Rabbit/IgG, supplier: Cell Signaling Technologies), catalog number: 85322S, dilution:(1:1000)
- anti-SUZ12 Rabbit/IgG, supplier: Cell Signaling Technologies), catalog number: 3737S, dilution:(1:1000)
- anti-Aurora A Rabbit/IgG, supplier: Cell Signaling Technologies), catalog number: 14475T, dilution:(1:500)
- anti-H3K27me3 Rabbit/IgG, supplier: Cell Signaling Technologies), catalog number: 9733S, dilution:(1:1000)
- anti- PLK1 Mouse/IgG, supplier: Biolegend, catalog number: B290751, dilution:(1:500)
- anti- EZH2 Mouse/IgG, supplier: BD Transduction Laboratory, catalog number: 612667, dilution:(1:1000)
- Anti-Rabbit IgG (H+L), HRP Conjugate, supplier: Promega, catalog number: W4011, dilution:(1:5000)
- Anti-Mouse IgG (H+L), HRP Conjugate, supplier: Promega, catalog number: W4021, dilution:(1:5000)

Validation

anti-GAPDH (Mouse/IgG Human) https://www.scbt.com/it/p/gapdh-antibody-0411
anti-AR (Mouse/IgG Human, Mouse, Rat) https://www.scbt.com/it/p/ar-antibody-441
anti-DNMT3A (Mouse/IgG Human, Mouse, Rat) https://www.scbt.com/p/dnmt3a-antibody-c-12
anti-DNMT1 (Rabbit/IgG Human, Mouse, Rat, Monkey) https://www.cellsignal.com/products/primary-antibodies/dnmt1-d63a6-xp-rabbit-mab/5032
anti-EED (Rabbit/IgG Human, Mouse, Rat, Monkey) https://www.cellsignal.com/products/primary-antibodies/eed-e4l6e-xp-rabbit-mab/85322
anti-SUZ12 (Rabbit/IgG Human, Mouse, Rat, Monkey) https://www.cellsignal.com/products/primary-antibodies/suz12-d39f6-xp-rabbit-mab/3737
anti-Aurora A (Rabbit/IgG Human) https://www.cellsignal.com/products/primary-antibodies/aurora-a-d3e4q-rabbit-mab/14475
anti-H3K27me3 (Rabbit/IgG Human, Mouse, Rat, Monkey) https://www.cellsignal.com/products/primary-antibodies/tri-methyl-histone-h3-lys27-c36b11-rabbit-mab/9733
anti- PLK1 (Mouse/IgG Human, Mouse) https://www.biolegend.com/ja-jp/products/purified-anti-plk-1-antibody-2929
anti- EZH2 (Mouse/IgG Human) https://www.bdbiosciences.com/en-us/products/reagents/microscopy-imaging-reagents/immunofluorescence-reagents/purified-mouse-anti-ezh2.612667
Anti-Rabbit IgG (H+L), HRP Conjugate https://ita.promega.com/products/protein-detection/primary-and-secondary-antibodies/anti-rabbit-igg-h-and-l-hrp-conjugate/?catNum=W4011
Anti-Mouse IgG (H+L), HRP Conjugate https://ita.promega.com/products/protein-detection/primary-and-secondary-antibodies/anti_mouse-igg-h-and-l-hrp-conjugate/?catNum=W4021

Eukaryotic cell lines

Policy information about cell lines

Cell line source(s)

PC3, DU-145, 22rV1, MDA-PCa-2b, LAPC-4, LNCaP, VCaP, and HEK 293T cell lines were purchased from ATCC (American Tissue Culture Collection) (Manassas, USA). The LAPC-4 cell line was a gift from Prof. Helmut Klocker, the LNCaP-abl cell line was a gift from Prof. Myles Brown (DFCI, Boston), and the THPl cell line was a gift from Prof. Saverio Minucci {IEO, Milan, original source: ATCC).

Authentication

The cell lines were tested by ATCC using a polyphasic (genotypic and phenotypic) testing to confirm identity.

Mycoplasma contamination

The supernatant of all cell lines was routinely tested (two times per month) using the MycoAlertTM Mycoplasma Detection Kit (Catalog #: LT07-318 Lonza). All cell lines show a ratio ranging between 0.3-0.7.

Commonly misidentified lines
(See ICLAC register)

No commonly misidentified cell lines were used in the study.

Animals and other organisms

Policy information about studies involving animals; ARRIVE guidelines recommended for reporting animal research

Laboratory animals	All in vivo studies were used 6-8 weeks old male NRG (NOD-Rag1null IL2rgnull, NOD rag gamma) mice.<br>Housing conditions: Before experimental procedures, mice were housed in individually vented cages, maintained at room temperature (20-22'C) and a 12h daylight cycle. Groups of 5 mice were kept in individuai cages of approx. 465 cm2. The cages were sealed, autoclaved before use, and used in a 'Sealsafe' rack (Techniplast) with a 0.2-micron aerosol bacteri a barrier vent. AII manipulation of the cages (e.g., to replace bedding) occured in a cage changing station (CCS, Techniplast), designed to maintain animals in a sterile airflow environment. For experimental procedures, mice were housed in groups of 4-5 mice in approx-355 cm2 filter-topped cages, either on racks in a specified pathogen-free barrier facility. Cages and filters were autoclaved before use, and experimental procedures and manipulation of the cages occurred in a sterile laminar flow hood (Skan AG).
Wild animals	no wild animals were used in the study
Field-collected samples	no field collected samples were used in the study.
Ethics oversight	All animal experiments were carried out accordingly to protocol approved by the Swiss Veterinary Authority/Board (TI-42-2018 and TI-10-2010) and received approval by the ethical committee of the Institute of Oncology Research (IOR).

Note that full information on the approval of the study protocol must also be provided in the manuscript.

Human research participants

Policy information about studies involving human research participants

Population characteristics	EZH2 protein expression was analyzed on tissue microarray (TMA) including, matched primary and CRPC samples (n=33) (University of Bern). Population characteristics: Gender: all males; Age range: between 58 and 89; Average age: 75 years. No genotypic information available.
Recruitment	Samples derive from Tissue microarrays collected from a retrospective study.
Ethics oversight	All prostate cancer samples from human subjects derive from a retrospective study and were obtained under approval by the Ethics Committee of Northwestern and Central Switzerland (EKNZ, No EK/1311 and 2015/228).

Note that full information on the approval of the study protocol must also be provided in the manuscript.

Flow Cytometry

Plots

Confirm that:

☒ The axis labels state the marker and fluorochrome used (e.g. CD4-FITC).

☒ The axis scales are clearly visible. Include numbers along axes only for bottom left plot of group (a 'group' is an analysis of identical markers).

☒ All plots are contour plots with outliers or pseudocolor plots.

☒ A numerical value for number of cells or percentage (with statistics) is provided.

Methodology

Sample preparation	The THP-M9 and the THP-Ml macrophages were dissociated by trypsinization, resuspended PBSl/FSC, staining with surface marker antibodies cocktail, and analyzed by flow cytometry. The CD14+ cells were isolateci from the Buffy coat. The Buffy coat was mixed 1:2 with PBS. The mixture was then added 3:1<br>to Ficoll gradient (Invitrogen, 17-1440-03) and spin down at 1500 rpm for 25 min at RT (w/o brakes). The leucocyte ring was collected and washed with PBS. Cells CD14+ cells isolateci by positive selection using magnetic column system of the Miltenyi Biotec (anti-CD14 microbeads 130-050-02 and LS MACS column system 130-042-401 Miltenyi Biotec The CD14+ cells were polarized to M2-like macrophages (see details in the method) and use for the experiment in CSS, CSS 1 nM DHT, or CSS 1 uM GSK-126 fresh medium and LNCaP-conditioned medium (see methods) For phenotype analysis, M2-like macrophages were suspended in PBS/FCS and then stained for 30-45 min at room temperature with a cocktail of surface markers antibodies. For the staining was used anti-CD14-BV650, anti-CD80-PE, anti-CD163-PECy7, and anti-CD206-BV510 antibodies were (eBioscience). To discriminate live cells, 7-ADD was added to the celi suspension.
Instrument	Samples were acquired on a BD LSR-Fortessa flow cytometry (BD Biosciences)
Software	Data were analyzed using FlowJo software
Cell population abundance	The CD14+ monocytes cells from the buffy coat were isolateci after staining with anti-CD14 microbeads (130-050-021 Milthenyi Biotec) and positive selection using the LS MACS column system (130-042-401 Miltenyi Biotec). To increased the purity of the sample the column purification was performed 2 times. The purity of CD14+ cells was checked by flow cytometry analysis after staining with an anti-CD14-BV650 antibody (eBioscience). The purity was always > of 95%

Gating strategy

Flow cytometry strategy: analyses of expression CD80 or CD206 or CD163 surface marker on a macrophages population selected base of the size (FSC vs SSC) / single cells / live cells (7-ADD-negative cells)/ and CD14 + positivity (monocyte cells)

☒ Tick this box to confirm that a figure exemplifying the gating strategy is provided in the Supplementary Information.
